# The Subcellular Distribution of Ryanodine Receptors and L-Type Ca^2+^ Channels Modulates Ca^2+^-Transient Properties and Spontaneous Ca^2+^-Release Events in Atrial Cardiomyocytes

**DOI:** 10.3389/fphys.2018.01108

**Published:** 2018-08-14

**Authors:** Henry Sutanto, Bart van Sloun, Patrick Schönleitner, Marc A. M. J. van Zandvoort, Gudrun Antoons, Jordi Heijman

**Affiliations:** ^1^Department of Cardiology, CARIM School for Cardiovascular Diseases, Maastricht University, Maastricht, Netherlands; ^2^Department of Physiology, CARIM School for Cardiovascular Diseases, Maastricht University, Maastricht, Netherlands; ^3^Department of Genetics and Cell Biology, Maastricht University, Maastricht, Netherlands

**Keywords:** calcium, sarcoplasmic reticulum, ryanodine receptor, spontaneous calcium releases, atrial fibrillation, subcellular distribution, computational modeling

## Abstract

Spontaneous Ca^2+^-release events (SCaEs) from the sarcoplasmic reticulum play crucial roles in the initiation of cardiac arrhythmias by promoting triggered activity. However, the subcellular determinants of these SCaEs remain incompletely understood. Structural differences between atrial and ventricular cardiomyocytes, e.g., regarding the density of T-tubular membrane invaginations, may influence cardiomyocyte Ca^2+^-handling and the distribution of cardiac ryanodine receptors (RyR2) has recently been shown to undergo remodeling in atrial fibrillation. These data suggest that the subcellular distribution of Ca^2+^-handling proteins influences proarrhythmic Ca^2+^-handling abnormalities. Here, we employ computational modeling to provide an in-depth analysis of the impact of variations in subcellular RyR2 and L-type Ca^2+^-channel distributions on Ca^2+^-transient properties and SCaEs in a human atrial cardiomyocyte model. We incorporate experimentally observed RyR2 expression patterns and various configurations of axial tubules in a previously published model of the human atrial cardiomyocyte. We identify an increased SCaE incidence for larger heterogeneity in RyR2 expression, in which SCaEs preferentially arise from regions of high local RyR2 expression. Furthermore, we show that the propagation of Ca^2+^ waves is modulated by the distance between RyR2 bands, as well as the presence of experimentally observed RyR2 clusters between bands near the lateral membranes. We also show that incorporation of axial tubules in various amounts and locations reduces Ca^2+^-transient time to peak. Furthermore, selective hyperphosphorylation of RyR2 around axial tubules increases the number of spontaneous waves. Finally, we present a novel model of the human atrial cardiomyocyte with physiological RyR2 and L-type Ca^2+^-channel distributions that reproduces experimentally observed Ca^2+^-handling properties. Taken together, these results significantly enhance our understanding of the structure-function relationship in cardiomyocytes, identifying that RyR2 and L-type Ca^2+^-channel distributions have a major impact on systolic Ca^2+^ transients and SCaEs.

## Introduction

Despite the significant advances in the treatment of cardiovascular diseases during the past 50 years, the frequency of cardiac arrhythmias, particularly atrial fibrillation (AF), is projected to increase, placing a significant burden on modern healthcare systems (Chugh et al., 2014; Roth et al., 2015; Morillo et al., 2017). Ca^2+^-handling abnormalities play a key role in ectopic activity and reentry, the two major arrhythmogenic mechanisms underlying AF (Heijman et al., 2014, 2016a; Landstrom et al., 2017). Dysfunctional ryanodine receptor type-2 (RyR2) channels, and/or SR Ca^2+^ overload can promote the occurrence of spontaneous sarcoplasmic reticulum (SR) Ca^2+^-release events (SCaEs) (Heijman et al., 2016a), which transiently increase the cytosolic Ca^2+^ concentration, activating the Na^+^/Ca^2+^-exchanger type-1 (NCX1), resulting in a depolarizing transient-inward current and promoting delayed after depolarizations (DADs) and triggered activity. Although potential proarrhythmic effects of changes in RyR2 expression and phosphorylation have been extensively discussed (Dobrev and Wehrens, 2014; Houser, 2014), these studies have generally employed tissue homogenates, ignoring the subcellular structure of Ca^2+^-handling proteins. However, there is increasing evidence that differences in subcellular structure critically influence cardiomyocyte Ca^2+^-handling. For example, there are important structural differences between atrial and ventricular cardiomyocytes that affect Ca^2+^-handling, including a relative paucity of transverse T-tubular structures in atrial cardiomyocytes, resulting in a centripetal Ca^2+^ wave propagating from RyR2 opposing L-type Ca^2+^ channels (LTCC) at the sarcolemma to RyR2 in the cell center (Arora et al., 2017). On the other hand, there is increasing evidence for a role of axial tubules in atrial cardiomyocyte Ca^2+^-handling (McNutt and Fawcett, 1969; Kirk et al., 2003; Dibb et al., 2013; Yue et al., 2017). Axial tubules promote a faster Ca^2+^ release from the SR in the center of the cell, which is partly mediated by coupling of LTCC to hyperphosphorylated RyR2 surrounding axial tubules (Brandenburg et al., 2016). Moreover, this axial tubular system undergoes extensive remodeling during cardiovascular disease, e.g., proliferating in the presence of atrial hypertrophy (Brandenburg et al., 2016) or disappearing in mice with atrial-specific knock-out of NCX1 (Yue et al., 2017). AF-related remodeling of the RyR2 distribution has also been reported, with RyR2 cluster fragmentation and redistribution in sheep with AF, which was associated with increased Ca^2+^-spark frequency (Macquaide et al., 2015). However, the exact impact of the subcellular distribution of RyR2 and LTCC on cardiomyocyte Ca^2+^-handling remains largely unknown. It is currently experimentally challenging to study both (sub)cellular structure and functional Ca^2+^-handling in human atrial cardiomyocytes, as the former usually requires fixation of the cardiomyocyte for antibody staining. The perfect control and observability provided by computational modeling may help to overcome this challenge (Heijman et al., 2016b).

A number of ventricular cardiomyocyte models have been developed that are able to simulate local Ca^2+^-handling and SCaEs (Colman et al., 2017; Walker et al., 2017). Models of atrial subcellular Ca^2+^-handling, on the other hand, are relatively scarce (Heijman et al., 2016b). We recently developed a human atrial cardiomyocyte model with stochastic gating of RyR2 channels and both transverse and longitudinal compartmentation of Ca^2+^-handling. Our model has a simple cell-type specific subcellular structure with LTCC only present on the lateral membranes, reflecting the relative paucity of T-tubules in isolated atrial cardiomyocytes (Voigt et al., 2014). However, all currently available atrial and ventricular cardiomyocyte models assume a homogeneous distribution of Ca^2+^-handling proteins. Here, we hypothesized that changes in the subcellular distribution of RyR2 and LTCC may promote proarrhythmic SCaEs. We employed both confocal microscopy and computational modeling to study for the first time the impact of the subcellular distribution of RyR2 and LTCC on Ca^2+^-handling in atrial cardiomyocytes.

## Materials and methods

A detailed overview of the experimental and computational methods can be found in the online Data Supplement. A brief summary is given below.

### Animal model, cardiomyocyte isolation, and confocal imaging

This investigation conformed with the Guide for the Care and Use of Laboratory Animals Published by the US National Institutes of Health (NIH Publications No. 85-23, revised 1996). All animal handling conformed with directive 2010/63/EU and experimental protocols were approved by the local ethical committee (DEC2014-112). New Zealand white rabbits (2.5–3.5 kg) were anesthetized and hearts were rapidly removed, excised, washed, perfused and cut into smaller pieces, as previously described (Greiser et al., 2014). Atrial cardiomyocytes were seeded on laminin coated coverslips. RyR2s were labeled with primary (mouse monoclonal (C3-33), IgG1, Sigma-Aldrich®, MO, 1:50) and secondary (Alexa® 488, goat anti-mouse, Abcam, UK, 1:100) antibodies (Dyba et al., 2003; Muller et al., 2012). The RyR2-stained atrial cardiomyocytes were imaged with a Leica TCS SP8 confocal microscope using a 63x objective (NA 1.40, oil immersion). The RyR2-Alexa® 488 antibody complex was detected at 420-520 nm under 488 nm laser illumination. Z-stacks were taken with a step size of 0.26 μm and an xy-resolution of 0.07 μm.

### Image processing

Image processing was employed to enable simulation with the experimental data (Supplemental Figure 1). The raw z-stacks were deconvolved using Huygens Professional (SVI, Netherlands). A single slice from the z-stack was selected and rotated to obtain a horizontal alignment of the RyR2. The image was thresholded and overlaid with a grid of ~1 μm^2^ units, in which the mean pixel intensity was calculated for every square of the grid as an indirect readout of local RyR2 density. The edges of the RyR2 expression matrix were detected and stretched to accommodate the rectangular dimensions of the virtual cardiomyocyte (100 × 18 μm). The resulting RyR2 distribution matrix was implemented in the computational model and employed for simulation.

### Computational modeling

Simulations were based on a previously published model of the human atrial cardiomyocyte in which local Ca^2+^-handling was simulated by dividing the virtual cardiomyocyte into 50 segments, each containing 18 subcellular Ca^2+^ domains located between two membrane domains (Voigt et al., 2014; Heijman et al., 2016b). The RyR2 expression in the published model was identical in every unit. We further optimized the 50-segment model with uniform RyR2 distribution based on experimental values (Tanaka et al., 2001; Woo et al., 2002; Kirk et al., 2003; Loughrey et al., 2004; Voigt et al., 2012, 2014; Greiser et al., 2014) of Ca^2+^-handling properties and SCaE properties reported in previous publications (Supplemental Figures 2, 3). In the present work, the model was extended to enable simulations with heterogeneous RyR2 distributions. Heterogeneity in RyR2 distribution across all 50 × 18 units was incorporated by drawing a random number from a Gaussian distribution with mean 1.0 and standard deviation σ for each unit and subsequently scaling these numbers to achieve the desired total RyR2 density. We also developed a model with a higher longitudinal resolution (100 segments), enabling simulation of the experimentally observed banded expression of RyR2. Axial tubules were simulated in the 100-segment model by including LTCC in axial-tubule-associated Ca^2+^ domains. Parameters of the 100-segment model with uniform RyR2 expression were adjusted to achieve similar Ca^2+^-handling properties as the 50-segment model (Supplemental Figures 2, 3) and heterogeneous RyR2 expression patterns were generated analogous to the 50-segment model. The values of the optimized parameters for both model versions are given in Supplemental Table 1. All results in the 100-segment model are provided following pacing for 250 beats at 0.5 Hz to achieve a quasi-steady state (Supplemental Figure 4). The source code for the model can be downloaded from the authors' website (http://www.jordiheijman.net).

### Data analysis and statistics

Due to the stochastic nature of the simulations, each condition was simulated at least 6 times and data were expressed as mean ± standard deviation. Statistical differences between conditions were evaluated using one- or two-way ANOVA with Tukey *post-hoc* test for multiple comparisons, or independent *t*-test, depending on the number of groups and the type of the data. Statistical analyses were performed using GraphPad Prism 7 (GraphPad Software Inc., La Jolla, CA).

## Results

### Effects of heterogeneity in RyR2 distribution on SCaEs

**Figure 1:**
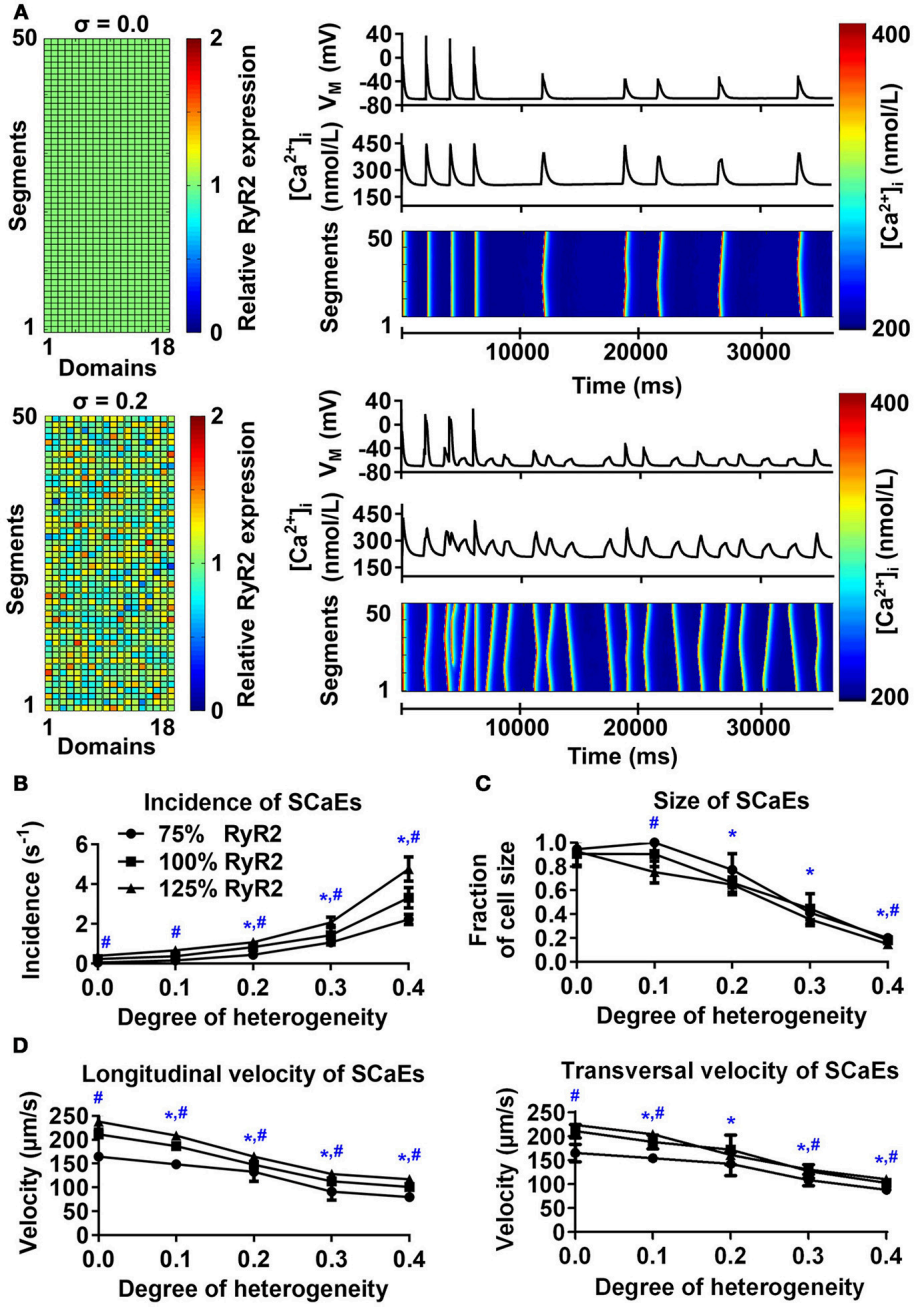
Effects of RyR2 distribution heterogeneity on spontaneous Ca^2+^-release events (SCaEs) in the 50-segment model. **(A)** Representative examples comparing heterogeneity (σ) of 0.0 (uniform expression, top) and 0.2 (bottom). The 50 × 18 matrices (left) show the relative RyR2 distribution. The membrane potential (V_M_), whole-cell Ca^2+^ concentration, and longitudinal line scan on the right show marked differences in number of SCaEs and corresponding delayed afterdepolarizations between both groups. **(B–D)** SCaE incidence **(B)** and size **(C)**, as well as longitudinal and transversal velocity of Ca^2+^ waves **(D)** as a function of RyR2 heterogeneity σ for different levels of total RyR2 expression (75% of control: circles; 100% of control: squares; 125% of control: triangles). SCaE incidence increases, while size decreases with increasing RyR2 heterogeneity. *indicates *P* < 0.05 vs. the group with heterogeneity 0.0 and ^#^indicates statistically significant differences among three levels of RyR2 expression; *n* = 6 per condition.

The model with uniform RyR2 distribution (σ = 0.0) produced a few large Ca^2+^ waves and corresponding DADs during follow-up after pacing at 0.5 Hz, in line with experimental data (Supplemental Figure 2). Increasing the heterogeneity of RyR2 distribution from σ = 0.0 to σ = 0.4 while keeping the total number of RyR2 constant substantially increased the incidence of SCaEs, but decreased their size (Figures 1A–C). For example, with σ = 0.4, the incidence of SCaEs was 14 × larger (3.31 ± 0.51 s^−1^ vs. 0.23 ± 0.04 s^−1^, *n* = 6, *p* < 0.05) and the average size of a Ca^2+^ wave as fraction of cardiomyocyte volume was 5x smaller than with σ = 0.0 (0.18 ± 0.02 vs. 0.91 ± 0.11, *n* = 6, *p* < 0.05). Increasing RyR2 heterogeneity also reduced the longitudinal (100.94 ± 3.66 μm/s vs. 211.24 ± 7.24 μm/s, *n* = 6, *p* < 0.05) and transversal (102.81 ± 3.94 μm/s vs. 210.79 ± 14 μm/s, *n* = 6, *p* < 0.05) velocity of Ca^2+^ waves. We compared the magnitude of the effect of altered RyR2 distribution to a 25% change in total RyR2 expression. In line with previous results (Voigt et al., 2014), increasing Ca^2+^ flux led to an increased number of SCaEs and smaller SCaE size (Figures 1B,C). Likewise, a 25% decrease in total RyR2 led to lower SCaE incidence and bigger SCaE size. Increasing RyR2 heterogeneity and total expression had synergistic effects on SCaE incidence.

**Figure 2:**
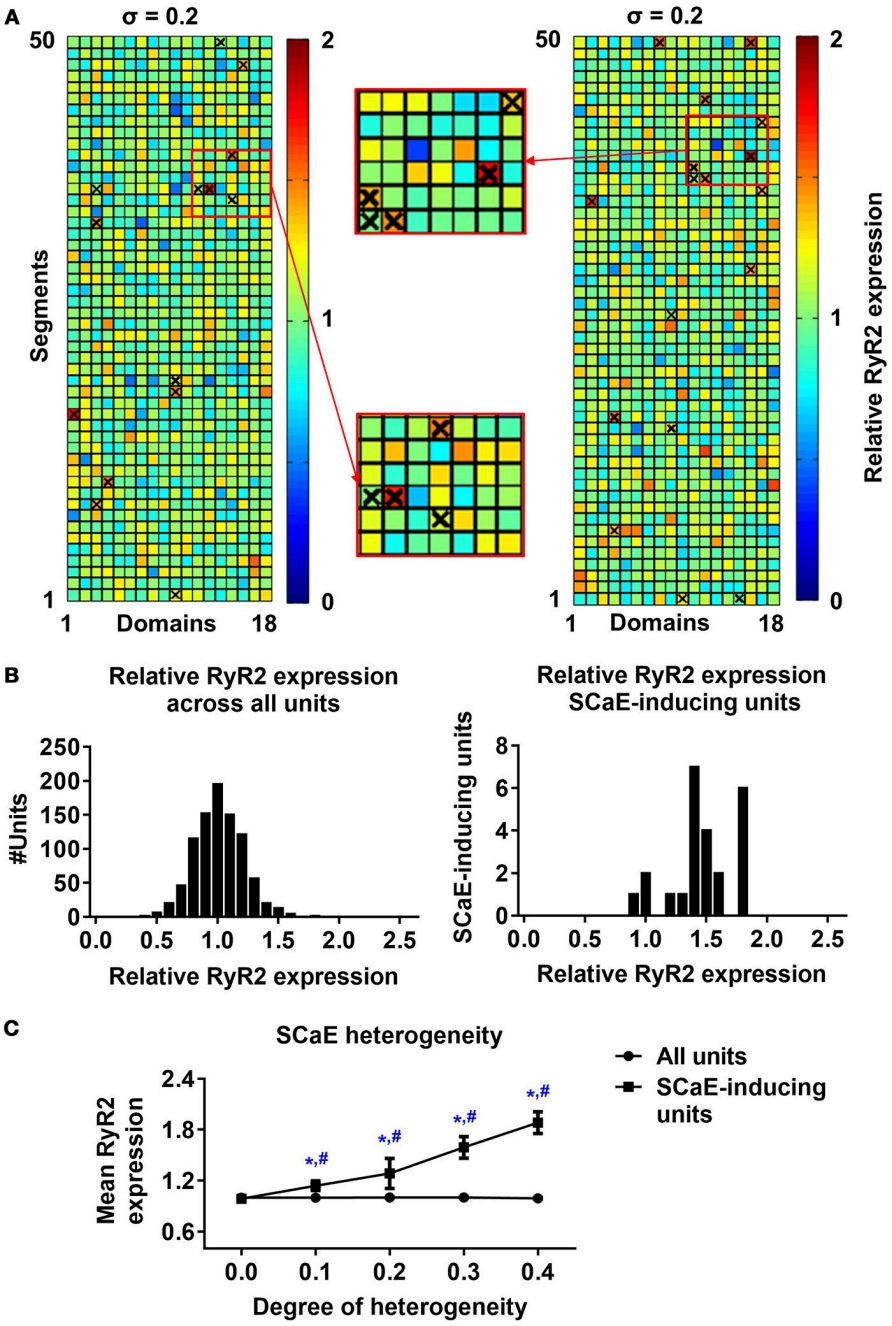
Origins of spontaneous Ca^2+^-release events (SCaEs). **(A)** Two representative examples of 50 × 18 matrices with heterogeneous RyR2 distribution (σ = 0.2). Red colors indicate high local RyR2 expression and blue colors low local RyR2 expression. The origins of individual Ca^2+^ waves are marked with crosses. Insets depict enlarged portions of the RyR2 distribution, showing that crosses mainly coincide with regions of high local RyR2 expression. **(B)** Histograms of relative RyR2 expression in all units (left) and units which were the origin of a SCaE (SCaE-inducing units). SCaEs arise mainly from units with high local RyR2 expression. **(C)** Mean relative RyR2 expression in SCaE-inducing units (squares) and all units (circles, 1.0 on average by definition) for different degrees of RyR2 heterogeneities. *indicates *P* < 0.05 vs. the group with heterogeneity 0.0 and ^#^indicates *P* < 0.05 between mean relative RyR2 expression in SCaE-inducing units and all units; *n* = 6 per condition.

Next, we investigated the origins of SCaEs in simulations with heterogeneous RyR2 distributions (crosses in Figure 2A). SCaEs preferentially arose from units with high local RyR2 expression. In agreement, comparison of the histograms of relative RyR2 expression of all 50x18 units with those of SCaE-inducing units revealed that SCaE-inducing units had significantly higher local RyR2 expression (Figure 2B). The difference in mean RyR2 expression between SCaE-inducing units and all units was most pronounced in simulations with large heterogeneity in RyR2 distribution (Figure 2C), establishing units with high local RyR2 expression as foci for SCaEs.

### Simulation of experimentally characterized RyR2 distributions

**Figure 3:**
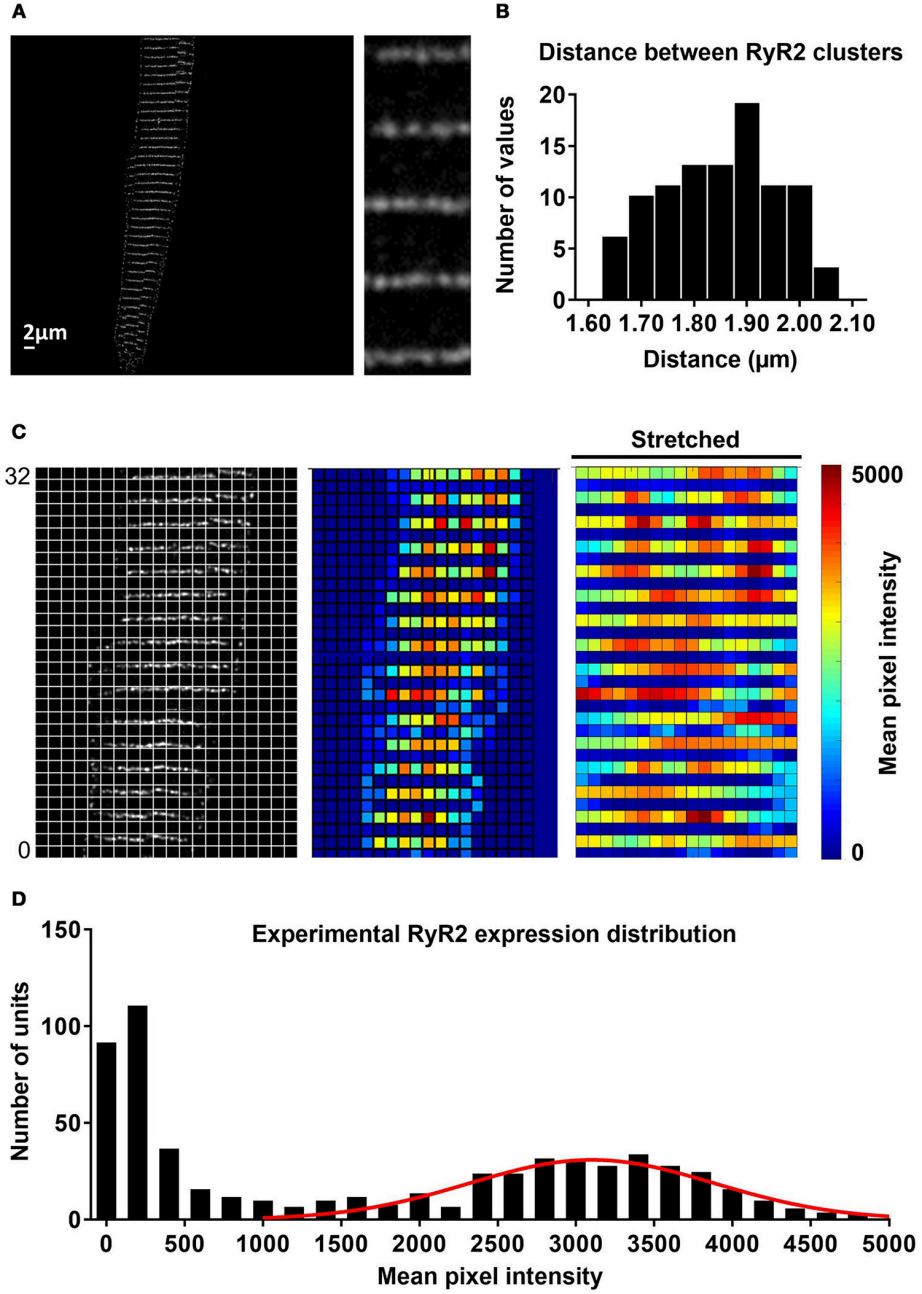
Experimental distribution of RyR2 expression in atrial cardiomyocytes. **(A)** Confocal image of RyR2 staining in a rabbit atrial cardiomyocyte. Inset shows region of interest at higher magnification, revealing that RyR2 clusters assemble in a regular banded pattern along the Z-band. **(B)** Distribution of distances between RyR2 bands, showing that majority of these gaps were around 1.8–1.9 μm. **(C)** Image processing of confocal images and alignment with a regular grid of ~1 μm^2^ units (left) enabled quantification of local RyR2 expression (right panel, red indicates high local RyR2 expression and blue low local RyR2 expression). **(D)** Histogram of experimental RyR2 expression distribution across all units with a large peak around 0, representing units between bands of RyR2 expression and a normal distribution of RyR2 expression within bands (line shows normal distribution with standard deviation of 0.253).

**Figure 4:**
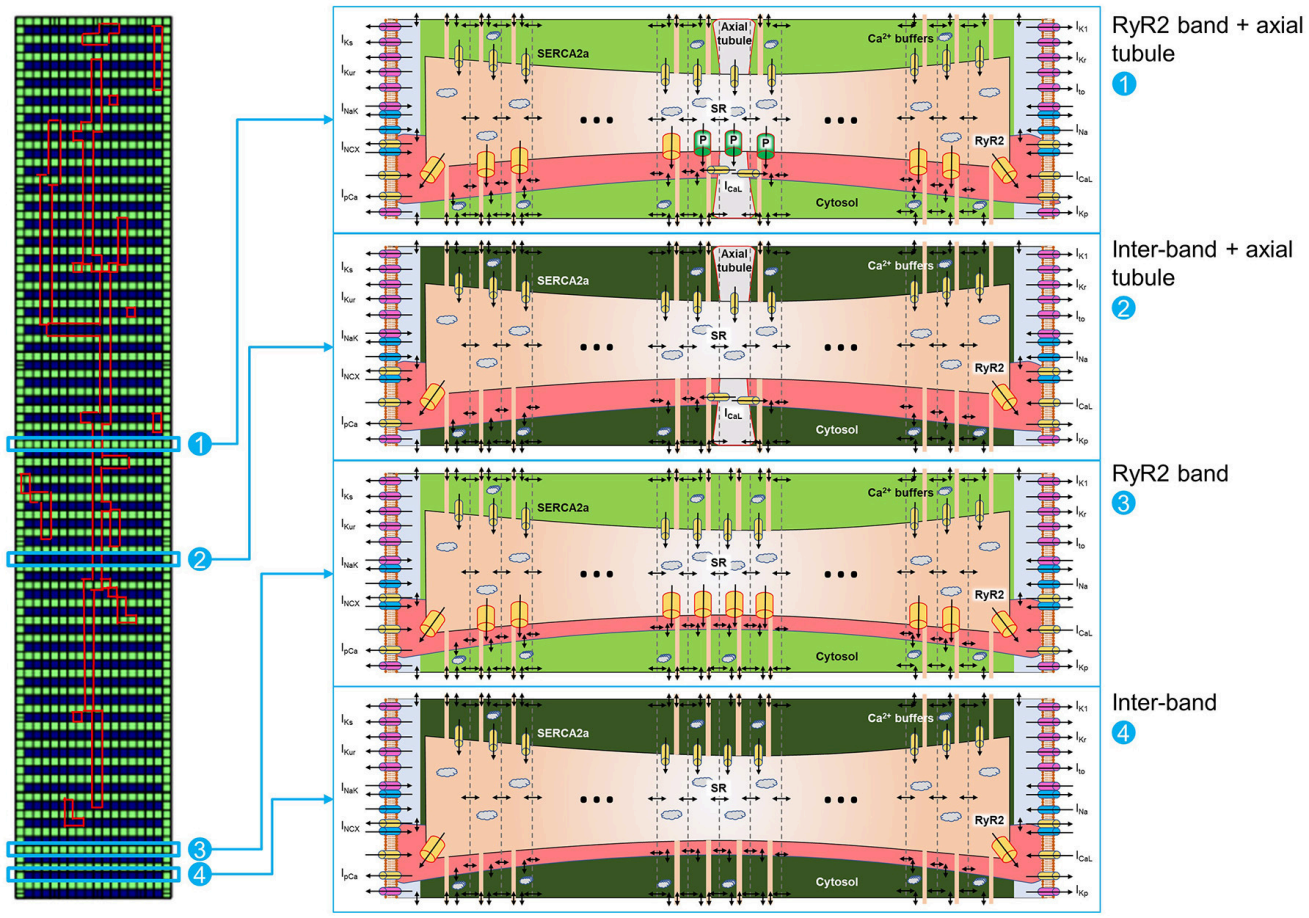
Schematic representation of 100-segment model. The model is represented by four different locations in the virtual cardiomyocyte. **(1)** Represents an RyR2 band with axial tubule and hyperphosphorylated RyR2 (indicated with “P” in the scheme) adjacent to the axial tubule, as reported by Brandenburg et al. (2016). **(2)** Represents an inter-band gap with axial tubule. **(3)** Represents an RyR2 band without axial tubule. **(4)** Represents an inter-band gap without axial tubule. Each segment is divided into 18 domains, as depicted in the matrix on the left, but only a subset of domains is shown for clarity. Please note the expression of RyR2 in the first and last domains in the segments located between RyR2 bands [i.e., **(2)** and **(4)**].

RyR2 distributions were studied in rabbit atrial cardiomyocytes. In line with previous results, we observed a banded RyR2 pattern with ~2 μm inter-band distance (Figures 3A,B). We analyzed the average RyR2 intensity after image-processing of the confocal images based on a square grid with 1 μm^2^ units and identified a significant variation in RyR2 distribution along the bands (Figure 3C). The histogram of relative RyR2 expression showed a large peak at near-zero levels, reflecting the units between RyR2 bands and a normal distribution with standard deviation σ = 0.253 for the RyR2 intensity within the bands (Figure 3D). In order to simulate this physiological RyR2 distribution, we increased the resolution of our model, simulating 100 segments of 1 μm with alternating pattern of RyR2 expression (Supplemental Figure 5) and validated its Ca^2+^-handling properties based on experimental data (Supplemental Figures 2, 3). We also incorporated the option to simulate the regional expression of LTCC localized in axial tubules and the experimentally observed hyperphosphorylation of axial-tubule-associated RyR2 (discussed below). A schematic representation of the subcellular structure of the model is shown in Figure 4.

**Figure 5:**
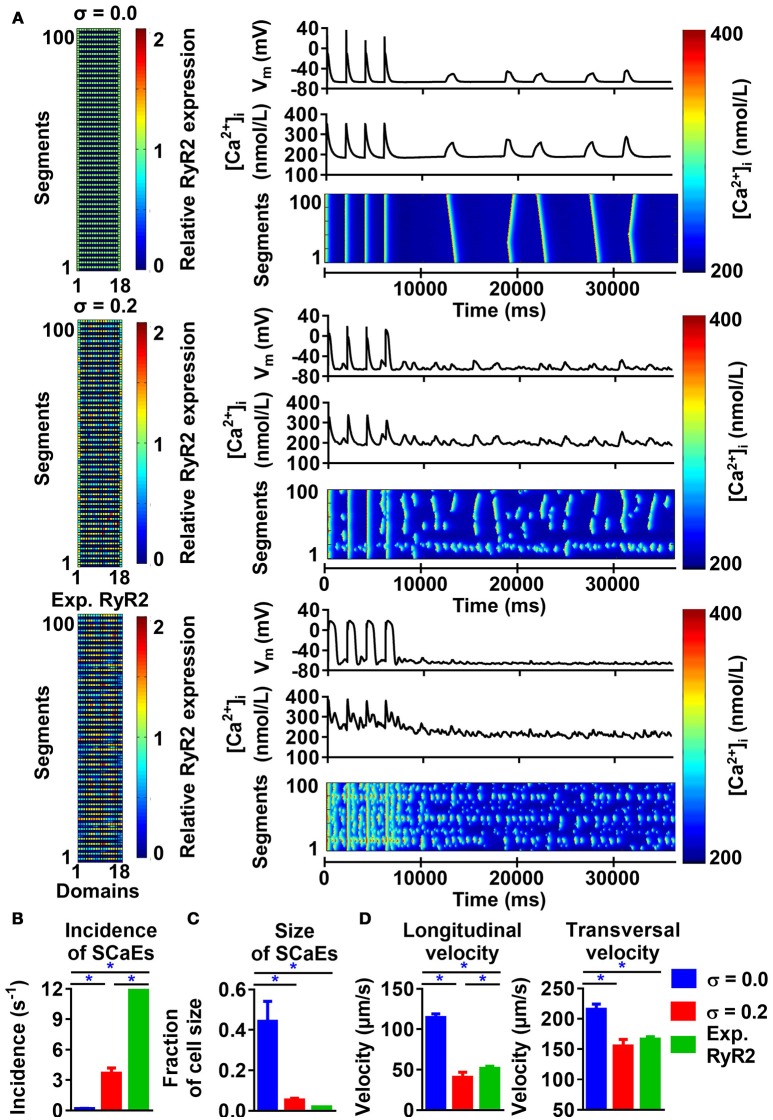
Effects of RyR2 distribution heterogeneity on spontaneous Ca^2+^-release events (SCaEs) in the 100-segment model. **(A)** Representative examples comparing heterogeneity (σ) of 0.0 (uniform expression, top), 0.2 (middle), and experimentally observed RyR2 expression patterns (bottom) on membrane potential (V_M_), whole-cell Ca^2+^-transient, and longitudinal line scan (right panels). The 100 × 18 matrices (left) of relative RyR2 expression incorporate the experimentally observed 2 μm inter-band distance. **(B–D)** SCaE incidence **(B)**, size **(C)**, as well as longitudinal and transversal velocity of Ca^2+^ waves **(D)** in 100-segment simulations with σ = 0.0, σ = 0.2, and experimentally observed RyR2 expression. Incorporation of the experimentally observed RyR2 expression in the original 100-segment model results in a large (non-physiological) number of small SCaEs. *indicates *P* < 0.05 between indicated groups; *n* = 6 per condition.

Similar to the 50-segment model, increasing heterogeneity of RyR2 expression in the 100-segment model led to an increase in SCaE incidence and reduction in SCaE size, longitudinal and transversal velocity (Figure 5), with SCaEs originating from units with high local RyR2 expression (Supplemental Figure 6). However, despite similar SCaE-incidence with a uniform RyR2 distribution, the increase in SCaEs with increasing RyR2 heterogeneity was more pronounced in the 100-segment model (115-fold vs. 14-fold increase from σ = 0.0 to σ = 0.4 in the 100-segment and 50-segment models, respectively; Supplemental Figure 7, Figure 1). Implementation of the experimentally observed RyR2 distribution pattern in the 100-segment model resulted in the occurrence of many small SCaEs, similar to our 100-segment simulations with heterogeneity (σ) 0.3 and 0.4 (with SCaE incidence of 12.96 ± 0.28 s^−1^ in the experimentally observed RyR2 distribution vs. 9.88 ± 1.16 s^−1^ in σ = 0.3 and 21.83 ± 2.62 s^−1^ in σ = 0.4, Figure 5B and Supplemental Figure 7). Longitudinal and transversal velocity of SCaEs in the experimentally observed RyR2 distribution model were similar to 100-segment simulations with σ = 0.2 (Figure 5D). Heterogeneities in RyR2 expression did not affect properties of the LTCC-triggered Ca^2+^ transient in the absence of preceding SCaEs (Supplemental Figure 8).

### Modulation of SCaE propagation by the distance between RyR2s and RyR2 clusters at the lateral membrane

**Figure 6:**
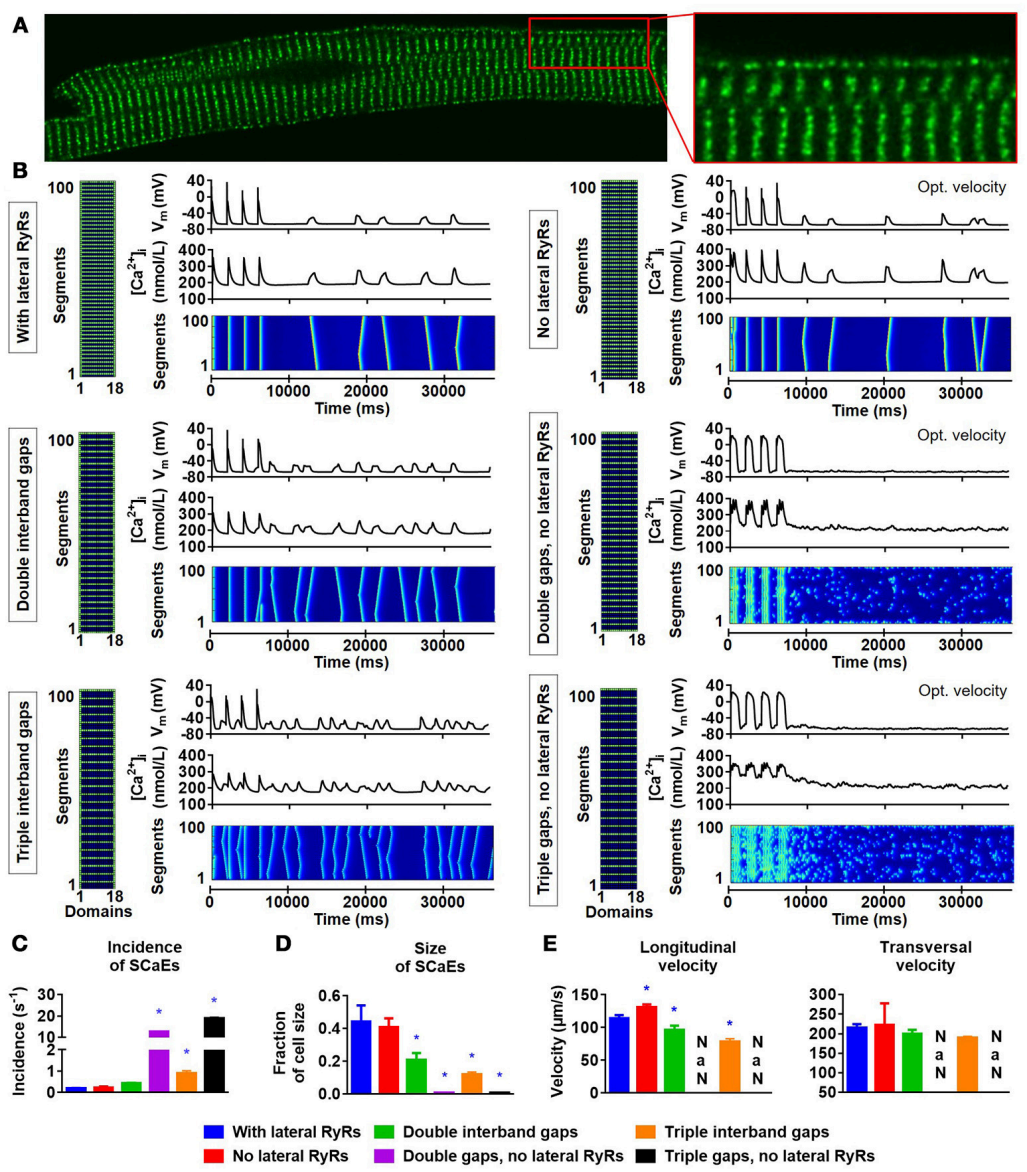
Lateral RyR2s, inter-band distance and the propagation of SCaEs. **(A)** Confocal image showing an increased density of RyR2 close to the lateral membrane in a rabbit atrial cardiomyocyte. **(B)** Representative examples of membrane potential (V_M_), whole-cell Ca^2+^-transient, and longitudinal line scan for the 100-segment model with single (~2 μm; top row), double (middle row) or triple (bottom row) inter-band distance, with (left column) or without (right column) expression of lateral RyR2s between bands. In all panels, the total number of RyR2 was adjusted to achieve a density of 2,772 RyR2 per unit. The time constant of longitudinal Ca^2+^ diffusion between SR release spaces was adjusted from 0.22 to 0.07 ms in the model without lateral RyR2 expression to obtain similar SCaE properties for physiological inter-band distances. **(C-E)** SCaE incidence **(C)**, size **(D)**, as well as longitudinal and transversal velocity of Ca^2+^ waves **(E)** for the six model versions. An increased inter-band distance increased the incidence of SCaEs, reduced their size and altered the longitudinal velocity without affecting the transversal velocity. *indicates *P* < 0.05 vs. the control model (100-segment model of single inter-band distance with lateral RyRs; blue bars), *n* = 6 per condition.

Previous studies have reported a closer spacing between RyR2s around the lateral membrane in rat atrial and ventricular cardiomyocytes (Chen-Izu et al., 2006; Galice et al., 2018), rabbit atrial (Musa et al., 2002), ventricular (Musa et al., 2002; Dan et al., 2007) and SA nodal cells (Musa et al., 2002), and human atrial cardiomyocytes (Brandenburg et al., 2016). We similarly observed a higher density of RyR2 clusters at the lateral membrane in rabbit atrial cardiomyocytes (Figure 6A) and accordingly incorporated RyR2 expression in the first and last Ca^2+^ unit of every segment of the virtual cardiomyocyte for the simulations of Figure 5. Next, we employed the model to assess the impact of these lateral RyR2s by comparing simulations with and without RyR2 in the outer Ca^2+^ units for segments located between RyR2 bands. The absence of RyR2s in the lateral region of the cardiomyocyte prevented the propagation of SCaEs, resulting in a reduced longitudinal velocity and numerous small SR Ca^2+^ releases (Supplemental Figure 9A). This behavior could be fully restored by reducing the time constant of Ca^2+^ diffusion between segments and normalizing the total RyR expression (SCaE incidence of 0.24 ± 0.04 s^−1^ in the group with normalized RyR2 expression and corrected longitudinal velocity vs. 0.19 ± 0.02 s^−1^ in the control group, *n* = 6, *p* > 0.05; Supplemental Figure 9B).

Next, we investigated the effect of alterations in the distance between RyR2 bands by simulating two or three segments without RyR2 expression between bands (instead of one). Although an increased RyR2 inter-band distance indeed slowed down the longitudinal velocity of SCaEs, it surprisingly did not impair propagation of SCaEs and resulted in an increased SCaE incidence (Figure 6B, left column; Figures 6C–E). Subsequent analyses showed that the RyR2 expressed at the lateral membrane in segments without RyR2 bands are critical for the propagation of SCaEs. In particular, a similar increase in RyR2 inter-band distance in the model without lateral RyR2 between bands (but with corrected longitudinal velocity of SCaEs at baseline) resulted in failure of Ca^2+^-wave propagation and many fragmented SCaEs (Figure 6B, right column). These data strongly suggest that the closer spacing of RyR2 at the lateral membrane that we observed experimentally may provide a safety factor for synchronous SR Ca^2+^ release but may also facilitate propagation of large proarrhythmic Ca^2+^ waves.

### Inter-band RyR2 expression and SCaEs

**Figure 7:**
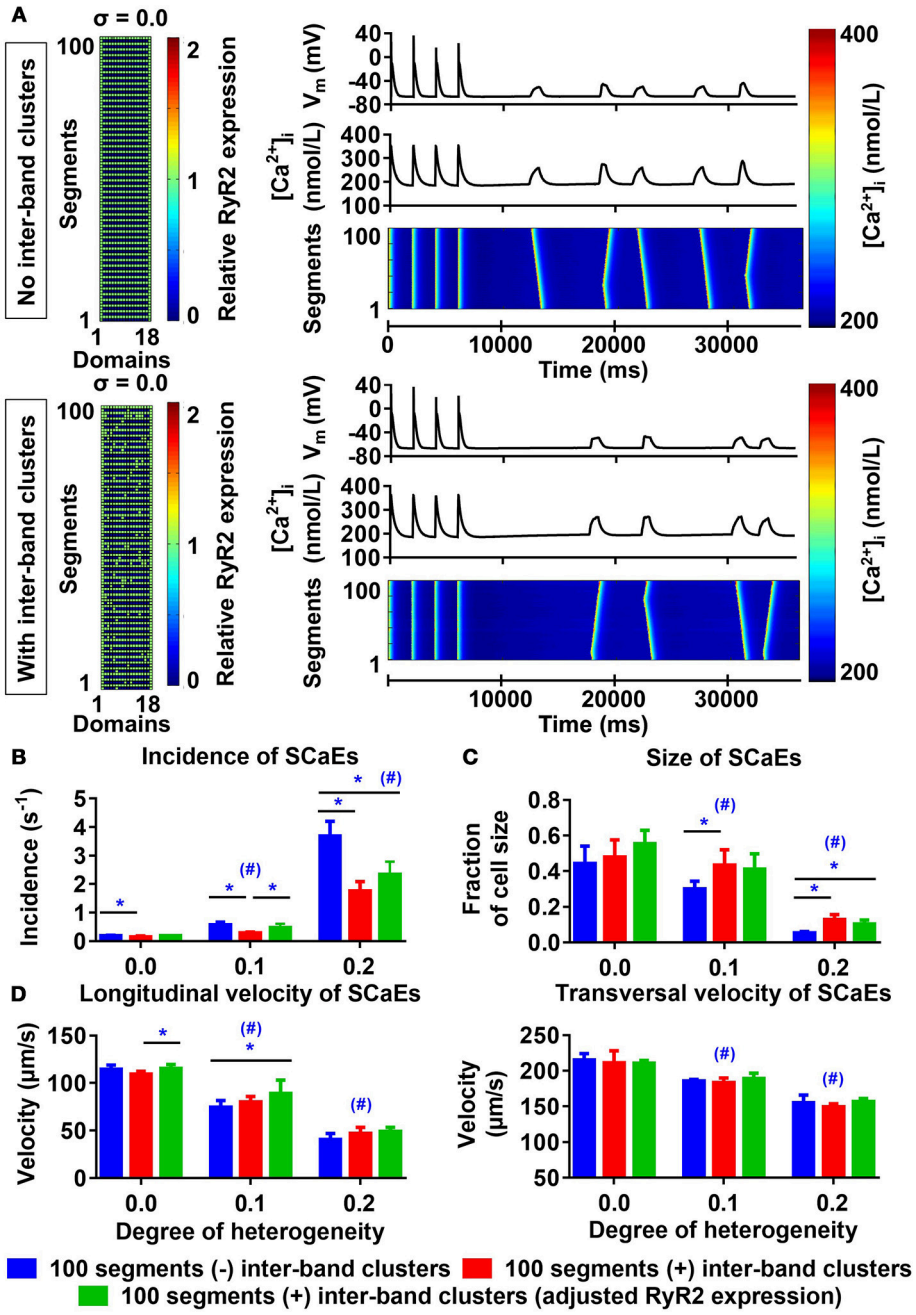
Effects of inter-band RyR2 clusters on spontaneous Ca^2+^ waves (SCaEs). **(A)** Comparison of RyR2 expression matrix (left), membrane potential (V_M_), whole-cell Ca^2+^ transient, and longitudinal line scan (right, top to bottom) in the model without inter-band RyR2 clusters (top) and with inter-band clusters (bottom) with uniform RyR2 expression (σ = 0.0). **(B–D)** SCaE incidence **(B)** and size **(C)**, as well as longitudinal and transversal velocity of Ca^2+^ waves **(D)** as a function of RyR2 heterogeneity σ (0.0–0.2) in the absence of inter-band RyR2 clusters (blue), in the presence of 10% inter-band RyR2 clusters (red), or in the presence of 10% inter-band RyR2 clusters with increased global RyR2 expression to ensure similar RyR2 density within the bands (green). *indicates *P* < 0.05 between groups and ^#^indicates *P* < 0.05 between a given level of heterogeneity and the group with heterogeneity 0.0; *n* = 6 per condition.

Similar to the results described by Macquaide et al. (2015), our confocal images also showed occasional RyR2 expression between individual bands. We employed the 100-segment model to better understand the functional effects of these inter-band RyR2 clusters. For any degree of heterogeneity, the presence of inter-band RyR2 clusters resulted in fewer, slightly larger SCaEs compared to simulations without inter-band clusters (Figures 7A–C, compare blue and red bars), without affecting longitudinal and transversal velocity of SCaEs (Figure 7D). We hypothesized that the reduction in SCaEs was due to a reduction in maximal local RyR2 expression resulting from the redistribution of RyR2 from the bands to the inter-band clusters. RyR2 expression per unit was indeed lower in the homogeneous simulations with inter-band clusters (2,543 vs. 2,772 RyR2 per unit) and increasing the total RyR2 expression of the 100-segment model with inter-band clusters to 2,772 in all units containing RyR2 normalized SCaE incidence for low levels of RyR2 heterogeneity (0.185 ± 0.015 s^−1^ in the normalized RyR2 expression group vs. 0.191 ± 0.018 s^−1^ in the group without inter-band clusters (σ = 0.0), *p* > 0.05 and 0.470 ± 0.138 s^−1^ for normalized RyR2 expression vs. 0.565 ± 0.105 s^−1^ without inter-band clusters (σ = 0.1), *p* > 0.05; Figure 7B). For high RyR2 heterogeneities, SCaE incidence remained lower in the presence of inter-band RyR2 cluster, even after adjusting the total RyR2 expression. Under these conditions, SCaEs are frequent, suggesting that annihilation or merging of small SCaEs arising around inter-band clusters contributes to a lower incidence of observed SCaEs, even after adjusting RyR2 expression.

### The impact of axial tubules on atrial Ca^2+^-handling

**Figure 8:**
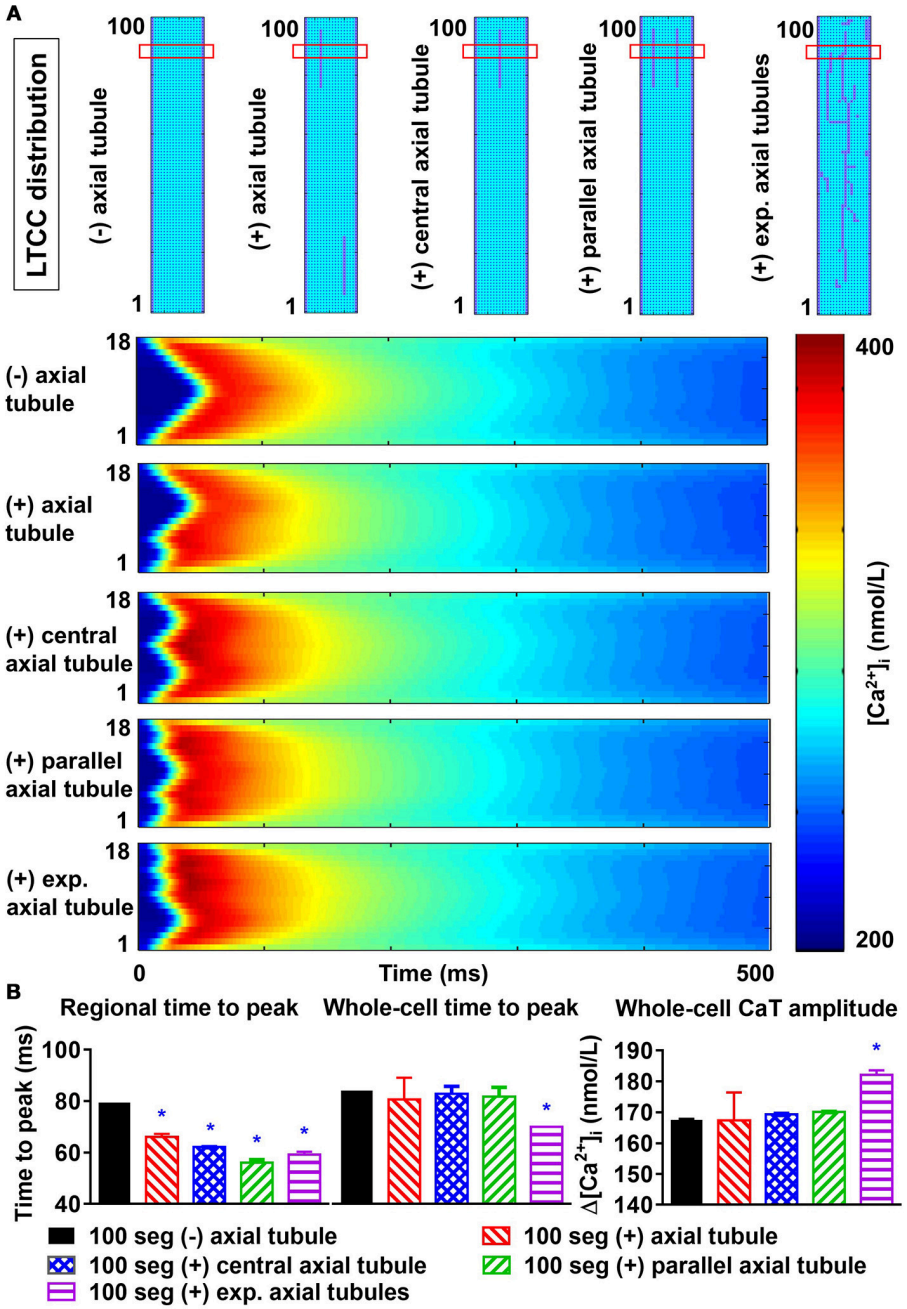
Effects of axial tubule(s) on centripetal Ca^2+^-wave propagation. **(A)** Schematic representations of model structure: no axial tubule (L-type Ca^2+^-channels [LTCC] only at lateral membranes), single axial tubule at 33% of cell width, single central axial tubule at 50% of cell width, two parallel axial tubules at 33 and 66% of cell width, or experimentally observed axial tubules based on Brandenburg et al. (2016) (top) and corresponding transversal line scans of Ca^2+^-induced Ca^2+^ release during an action potential, showing centripetal Ca^2+^-wave propagation (bottom). **(B)** Quantification of time to peak (left) and Ca^2+^-transient amplitude (right) for the indicated line scan in the four groups (“regional”) or for the whole-cell Ca^2+^ transient. Increasing numbers of axial tubules and more centrally located axial tubules decrease the time-to-peak and slightly increase the Ca^2+^-transient amplitude. The magnitude of the increase depends on the number of axial tubules. *indicates *P* < 0.05 vs. the 100-segment group without axial tubules (black bars); *n* = 6 per condition.

Axial tubules are unique to atrial cardiomyocytes, but their role in Ca^2+^-handling is not fully understood. Axial tubules contain LTCCs, modulating Ca^2+^-induced Ca^2+^ release and centripetal Ca^2+^-wave propagation. We incorporated various configurations of axial tubules in the 100-segment model and investigated their impact on depolarization-induced, LTCC-triggered Ca^2+^ transients and SCaEs. Addition of an axial tubule to the model reduced time-to-peak of the regional LTCC-triggered Ca^2+^ transient (based on a virtual transversal line scan through the region with the axial tubule) and slightly increased the Ca^2+^-transient amplitude (Figure 8). Moreover, time to peak of the transversal-line-scan-based Ca^2+^-transient is influenced by both the number of parallel axial tubules and their location. The reduction in time to peak was largest with an axial tubule located in the center of the virtual cardiomyocyte, and addition of two or more parallel axial tubules further reduced the time to peak (Figure 8). The impact of axial tubules on whole-cell Ca^2+^-transient properties depended on axial-tubule length and was limited for short axial tubules (compare regional and whole-cell time to peak in Figure 8B). However, a longer axial tubule network, such as that observed experimentally (Brandenburg et al., 2016), also significantly shortened time to peak of the whole-cell Ca^2+^ transient (Figure 8B, rightmost columns).

**Figure 9:**
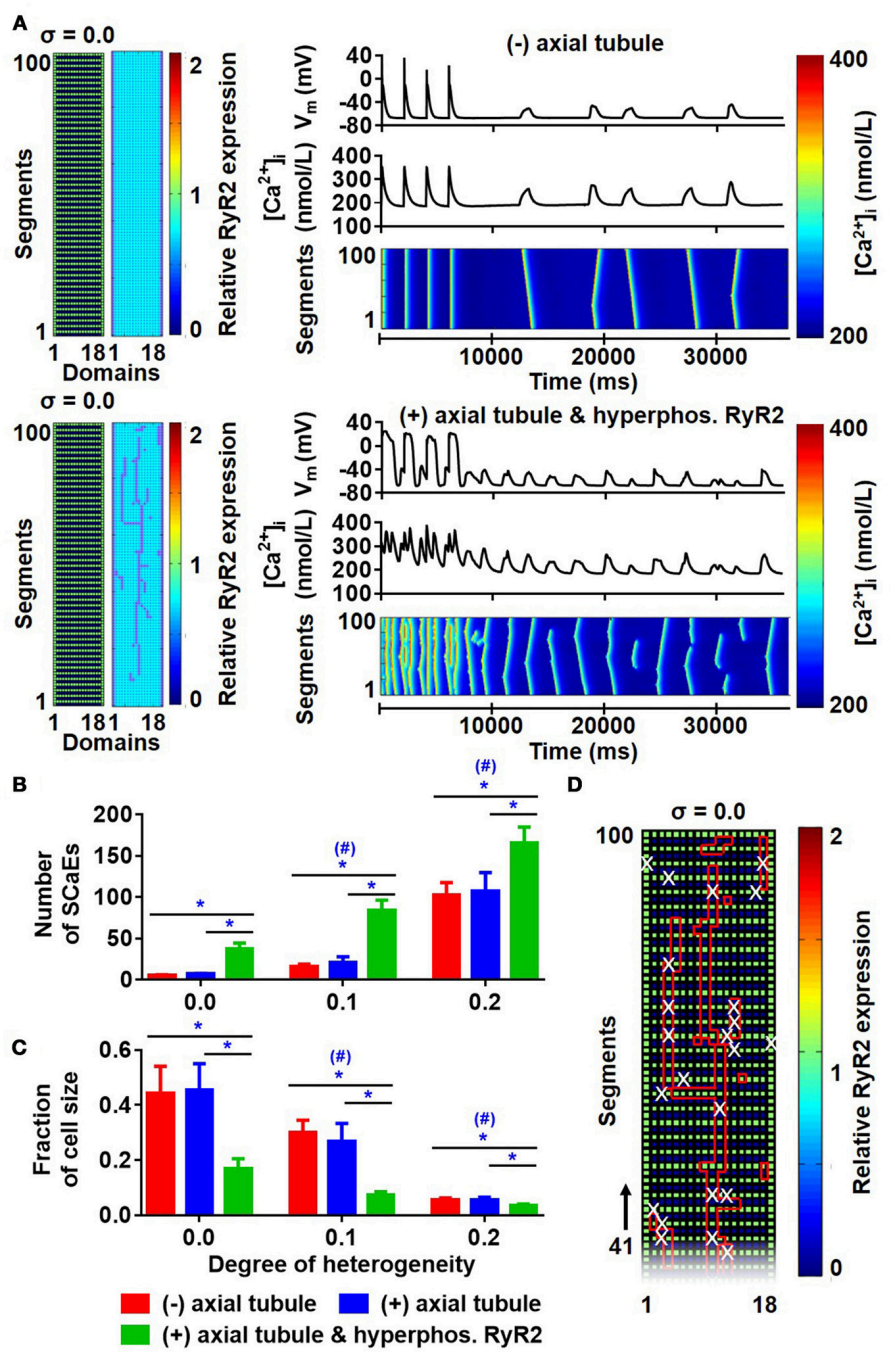
Effects of axial tubules on SCaEs. **(A)** Comparison between the model without axial tubules and model with axial tubules with uniform RyR2 expression (σ = 0.0), but hyperphosphorylated RyR2 around axial tubules. **(B,C)** Number of Ca^2+^ waves **(B)** and SCaE size **(C)** in the absence of axial tubules (red bars), presence of axial tubules (blue bar), or presence of axial tubules with hyperphosphorylation of surrounding RyR2 (green bars). **(D)** Distribution of RyR2 expression and location of axial tubules (red lines) in relation to the origin of SCaEs (white crosses), showing that SCaEs primarily originated from RyR2 clusters adjacent to the axial tubules. *indicates *P* < 0.05 between groups and ^#^indicates *P* < 0.05 between a given level of heterogeneity and the group with heterogeneity 0.0; *n* = 6 per condition.

There were no differences in the longitudinal Ca^2+^-wave velocity between simulations with and without axial tubules. Similarly, the presence of axial tubules with LTCC did not affect the number of SCaEs (Figures 9A,B; compare red and blue bars), consistent with the idea that SCaEs result from stochastic RyR2 openings that are independent of LTCCs. However, a recent study (Brandenburg et al., 2016) noted that although RyR2 expression adjacent to the axial tubule was not different from other parts of the cardiomyocyte, these RyR2 near axial tubules were hyperphosphorylated. We simulated RyR2 hyperphosphorylation in our model by increasing RyR2 open probability for all RyR2 located in units surrounding axial tubules. In the presence of hyperphosphorylated RyR2 surrounding axial tubules, the number of SCaEs was increased, with a corresponding reduction in their size (Figures 9B,C). Of note, SCaEs indeed primarily originated around the axial tubule (Figure 9D, Supplemental Figure 10), although in the presence of a heterogeneous RyR2 distribution, regions with high local RyR2 expression may also act as foci (Supplemental Figure 10).

### Predicting the effects of beta-adrenergic stimulation on SCaEs

Beta-adrenergic stimulation is an accepted promoter of SCaE-mediated triggered activity (Chen et al., 2014). Here, we investigated the functional consequences of three established downstream targets of beta-adrenergic stimulation. Chronic AF is associated with a hyperphosphorylation-mediated 100–500% increase in RyR2 open probability (Voigt et al., 2012). Therefore, we first implemented a 100% increase of RyR2 open probability to simulate the effect of beta-adrenergic stimulation. This resulted in smaller and more frequent SCaEs compared to baseline (incidence of 2.03 ± 0.09 s^−1^ in the increased RyR2 open probability group vs. 0.19 ± 0.02 s^−1^ in the baseline group, *n* = 6, *p* < 0.05; Supplemental Figure 11). Second, we implemented an increased SERCA2a affinity for cytosolic Ca^2+^ to reflect phospholamban phosphorylation. In the presence of increased SERCA2a function, the incidence of SCaEs was increased without any statistically significant change in the size of SCaEs (Supplemental Figure 12). In addition, an increase in longitudinal and transversal velocities of SCaEs was observed. Third, we implemented a homogenous increase of LTCC function by doubling the maximum conductance of the channel, which resulted in an increased SCaE incidence without any statistically significant changes in the size and velocities of SCaEs (Supplemental Figure 13). The combination of all three modifications, reflecting maximal beta-adrenergic stimulation, produced a dramatic increase in SCaEs (incidence of 27.77 ± 14.92 s^−1^ vs. 0.19 ± 0.02 s^−1^ in the baseline group, *n* = 6, *p* < 0.05), including proarrhythmic triggered activity (Supplemental Figure 14).

### A computational model incorporating physiological RyR2 and LTCC distributions

**Figure 10:**
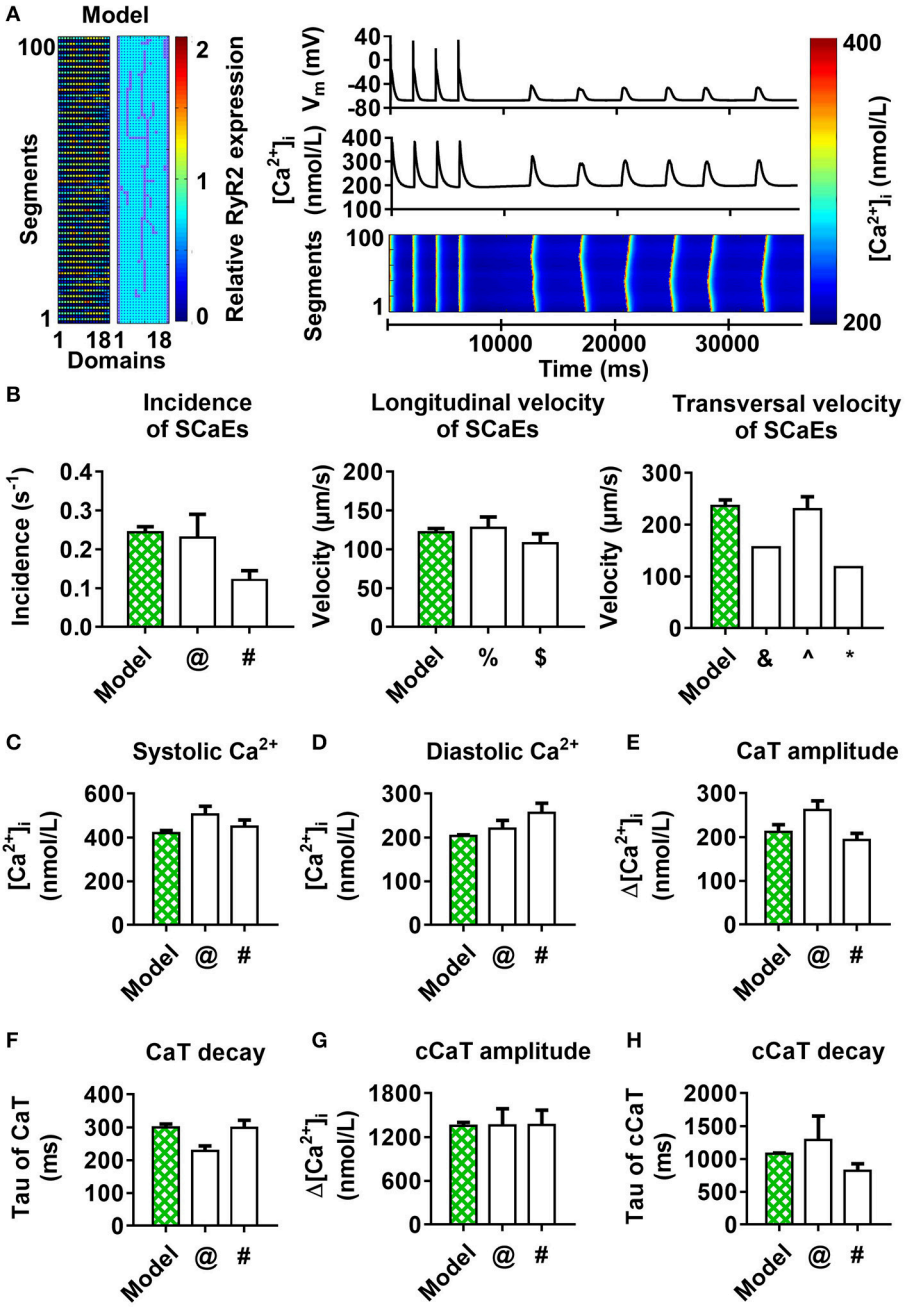
100-segment model with experimental RyR2 distribution and LTCC expression based on experimentally observed axial tubules, with RyR2 hyperphosphorylation around axial tubules. **(A)** Model structure (left panel) and longitudinal line scan of the optimized 100-segment model (“Model”). **(B–H)** Comparison of SCaE characteristics **(B)** and whole-cell Ca^2+^-transient properties **(C–H)** of the model to previously published experimental data ^$^Tanaka et al. (2001); ^∧^Woo et al. (2002); ^&^Kirk et al. (2003); ^%^Loughrey et al. (2004); ^@^Voigt et al. (2012); ^#^Voigt et al. (2014); ^*^Greiser et al. (2014).

Finally, we combined both the experimentally observed RyR2 distribution (Figure 3) and axial tubule network with LTCC in a novel model of the human atrial cardiomyocyte. When using the parameters established for the model with uniform RyR2 distribution and no axial tubules, this model showed a non-physiological number of small SCaEs, in line with the effects of the experimental RyR2 distribution in Figure 5A. As such, we performed a parameter optimization to reproduce the experimentally observed Ca^2+^-handling properties in the combined model (Figure 10).

## Discussion

Recent studies have identified a major role for cardiomyocyte Ca^2+^-handling abnormalities in cardiac arrhythmias and have provided insight into the underlying molecular mechanisms (Heijman et al., 2016a; Landstrom et al., 2017). However, the impact of the subcellular distribution of RyR2 and LTCC on cardiomyocyte Ca^2+^-handling remains largely unknown. It is currently experimentally challenging to study both (sub)cellular structure and functional Ca^2+^-handling in the same cardiomyocyte. Here, we extended a computational model of the human atrial cardiomyocyte with a more physiological subcellular structure, including heterogeneous RyR2 distributions and axial tubules with LTCC. Our computational analyses showed that increasing RyR2 heterogeneity resulted in more, smaller SCaEs arising from regions with high local RyR2 expression. LTCC located in axial tubules produced a faster, more synchronous CICR, which was modulated by the location and extent of the axial tubular network. Moreover, hyperphosphorylation of RyR2 surrounding axial tubules increased the incidence of SCaEs and DADs. Finally, we developed and validated a novel human atrial cardiomyocyte model with physiological RyR2 distribution and axial tubules with LTCC based on experimental observations, which can serve as a tool for future studies.

### Role of subcellular structure in cardiomyocyte Ca^2+^-handling

An increased SCaE incidence in atrial cardiomyocytes from patients with AF contributes to the initiation of DADs and cellular triggered activity (Hove-Madsen et al., 2004; Voigt et al., 2012, 2014), which, when occurring at the tissue level, may act as initiators of AF or may sustain the arrhythmia when occurring repetitively at high frequency (Vincenti et al., 2006; Voigt et al., 2014). SCaEs are promoted by increased SR Ca^2+^ load leading to store-overload-induced SR Ca^2+^ release (MacLennan and Chen, 2009). In agreement, SERCA2a activity is increased in patients with paroxysmal AF and is associated with increased SR Ca^2+^ load and a higher incidence of SCaEs (Voigt et al., 2014). In addition, RyR2 dysfunction can increase the incidence of SCaEs even in the absence of increased SR Ca^2+^ load. RyR2 mutations leading to catecholaminergic polymorphic ventricular tachycardia have been associated with familial AF (Enriquez et al., 2016) and genetic mouse models with these mutations show pronounced atrial Ca^2+^-handling abnormalities (Shan et al., 2012). Moreover, RyR2 open probability is increased, likely due to hyperphosphorylation of the RyR2 channel, in patients with long-standing persistent AF (Voigt et al., 2012). Besides changes in cardiomyocyte Ca^2+^-handling, AF produces structural remodeling at the level of the single cardiomyocyte (atrial cellular hypertrophy, myolysis, alterations in size and distribution of mitochondria and SR) (Morillo et al., 1995; Brundel et al., 2002; Goette et al., 2016), suggesting a potential role for changes in subcellular structure in regulating cardiomyocyte Ca^2+^-handling. Indeed, Macquaide et al. (2015) reported that ultrastructural reorganization of RyR2 clusters in atrial cardiomyocytes of sheep with persistent AF is associated with overactive Ca^2+^ release. In addition, Lenaerts et al. (2009) reported that in sheep with persistent AF, there was loss of T-tubule organization, fewer Ca^2+^ channel-RyR couplings, and reduced efficiency of the coupling at subsarcolemmal sites, which led to a reduction in SR Ca^2+^ release despite preserved SR Ca^2+^ content. Thus, there is substantial indirect evidence for a role of subcellular structure in cardiomyocyte Ca^2+^-handling. However, a systematic analysis of the impact of changes in Ca^2+^-handling protein distributions on whole-cell proarrhythmic Ca^2+^-handling is lacking.

Consistent with previous publications (Hiess et al., 2015; Yue et al., 2017), our confocal images of rabbit atrial cardiomyocytes showed a banded RyR2 expression with inter-band distance of ≈ 1.9 μm. Moreover, there appears to be a substantial heterogeneity of RyR2 density along the z-bands. We increased the spatial resolution of our computational model to enable simulations of the banded RyR2 pattern and incorporated the experimentally observed RyR2 distribution in our model. In line with the work by Macquaide et al. (2015), our simulations showed a pronounced impact of RyR2 distribution on SCaE incidence and size. Indeed, physiologically observed degrees of RyR2 heterogeneity had a larger impact on SCaEs than differences in total RyR2 expression observed between patients with sinus rhythm and paroxysmal AF (Voigt et al., 2014). Of note, our simulations identified regions with high local RyR2 expression as foci for SCaEs, which is in line with recent experimental work by Galice et al. (2018).

In ventricular cardiomyocytes, LTCC are primarily (but not exclusively, Best and Kamp, 2012) located in T-tubules, promoting synchronous SR Ca^2+^ release throughout the cell. Recent studies have identified a similar role for axial tubules in atrial cardiomyocytes (Dibb et al., 2013). For example, Brandenburg et al. (2016) reported the importance of axial tubules in atrial cardiomyocytes in maintaining Ca^2+^-handling and Ca^2+^-wave propagation to the center of atrial cardiomyocytes. Similarly, Yue et al. (2017) observed synchronous SR Ca^2+^ release in mouse atrial cells, which was ascribed to the presence of transverse-axial tubules. We employed the perfect control provided by computational models to study the exact effects of different locations, numbers, and distributions of such axial tubules on whole-cell Ca^2+^-handling. Incorporation of axial tubules produced a more synchronous SR Ca^2+^ release, as evident from a W-shaped instead of V-shaped pattern in simulated transversal line scans. The W-shaped patterns varied with different locations of the axial tubules, consistent with experimental findings (Kirk et al., 2003; Yue et al., 2017) noting that the presence and location of the transverse-axial tubular system determined the shape of the whole-cell Ca^2+^ transient and transversal Ca^2+^ waves. Our simulations showed that more centrally located and/or higher number of axial tubules reduced the local time-to-peak. However, in order to affect the global time-to-peak and global Ca^2+^-transient amplitude, the length of the axial tubules had to be sufficiently long to affect a large part of the virtual cardiomyocyte. Brandenburg et al. (2016) reported that instead of an increase in the number of RyR2 adjacent to the axial tubules, there was a selective local RyR2 hyperphosphorylation, which led to a faster Ca^2+^ release at axial tubule locations residing inside the cell. Our simulations showed that the presence of axial tubules *per se* did not affect SCaEs, but that simulation of local hyperphosphorylation of RyR2 increased the number of SCaEs. Furthermore, we confirmed that regions with local hyperphosphorylation acted as the origins of SCaEs. However, these results depended on the amount of RyR2 heterogeneity and the number of axial tubules, with SCaEs also arising from regions with high local RyR2 expression independent of RyR2 hyperphosphorylation. Taken together, these findings underline the importance of the subcellular distribution of Ca^2+^-handling proteins in atrial cardiomyocytes for cardiac arrhythmogenesis.

Previous publications (Musa et al., 2002; Chen-Izu et al., 2006; Dan et al., 2007; Brandenburg et al., 2016) have reported an increased RyR2 density at the lateral membrane using confocal microscopy. However, the role of these lateral RyR2 clusters remains unknown. Here, we employed a computational model with no expression of lateral RyR2s to investigate their role in the propagation of SCaEs. Our data show that lateral RyR2s hold a very important role as “bridges” that facilitate Ca^2+^-wave propagation. Removal of lateral RyR2 clusters impaired Ca^2+^-wave propagation and resulted in more, but smaller SCaEs, effectively converting proarrhythmic Ca^2+^ waves to Ca^2+^ sparks.

### Comparison to previous models

Several computational models have been developed to study cardiomyocyte Ca^2+^-handling abnormalities. These include, on the one hand, highly detailed models of a single Ca^2+^-release unit (CRU) to study the molecular determinants of SR Ca^2+^ release. For example, Hake et al. (2012) developed a computational model with a highly detailed, electron microscopy-based computational geometry of a CRU from a mouse ventricular cardiomyocyte to simulate local Ca^2+^ sparks. Walker et al. (2014) developed a detailed three-dimensional model of a CRU incorporating diffusion, intracellular buffering systems, and stochastically gated RyRs and LTCCs to simulate local Ca^2+^ dynamics with a high spatial resolution. This work showed that perturbations to subspace dimensions strongly alter Ca^2+^-spark dynamics. Similarly, Zahradnikova and Zahradnik (2012) constructed virtual CRUs composed of a variable number of RyRs distributed in clusters in line with the experimentally observed cluster-size distribution to provide a description of Ca^2+^-spark properties for spontaneous and triggered Ca^2+^ sparks. These studies strongly suggest that the organization of the CRU plays a critical role in determining the characteristics of microscopic Ca^2+^-release events (sparks) but have not simulated whole-cell Ca^2+^-handling abnormalities, which would be relevant to study arrhythmogenesis.

On the other hand, a number of models have been developed to study whole-cell Ca^2+^-handling: Walker et al. (2017) developed a biophysically detailed three-dimensional model of the ventricular cardiomyocyte with stochastic gating of RyR2 channels and determined the impact of cytosolic and SR Ca^2+^ concentrations, basal inward-rectifier K^+^ current density, and gap junction conductance on DADs and triggered activity using this model. Likewise, Wescott et al. (2016) developed a mathematical whole-cell model, incorporating realistic stochastic gating of LTCCs and RyRs to investigate excitation-contraction coupling and Ca^2+^-spark fidelity. Recently, Colman et al. (2017) developed a detailed three-dimensional multiscale model of a ventricular cardiomyocyte based on scanning electron microscopy data to examine the effects of a realistic SR structure on pro-arrhythmic Ca^2+^ dynamics, alternans, and SCaEs. Song et al. (2018) also investigated the influence of subcellular structure on Ca^2+^-handling in a model with a three-dimensional network of CRUs representing different transverse tubule network structures, including uniform and random distribution of transverse tubules, to investigate Ca^2+^ sparks, DADs, and triggered activities. However, these studies were primarily done in ventricular cardiomyocytes, which have well-established differences in subcellular structures, notably the configuration and number of axial tubules and a different composition of ion channels from atrial cardiomyocytes.

Here, we developed an atrial cardiomyocyte model with atrial-specific subcellular structure and electrophysiology that can simulate multiple physiological properties of cardiomyocyte Ca^2+^-handling, as well as proarrhythmic Ca^2+^-handling abnormalities. This model has an intermediate level of detail, incorporating heterogeneous distributions of Ca^2+^-handling proteins with micrometer resolution, a level that is of the same order of magnitude as the experimental information about the distributions obtained with confocal imaging. This level of detail is highly suitable to study the structural determinants of whole-cell Ca^2+^-handling abnormalities that are relevant for arrhythmogenesis. Moreover, because of its relatively modest computational complexity, this model can also be employed in future studies to investigate the determinants of triggered activity at the tissue level, something that is not possible with the previously developed, highly detailed, three-dimensional models.

### Potential limitations

Our model with local Ca^2+^-handling strongly suggested that the subcellular distribution of RyR2 and LTCC has a major impact on cardiomyocyte Ca^2+^-handling. However, the model only considered a 2-dimensional representation of the cardiomyocyte, equivalent to a single slice from a z-stack. Previous computational studies using simpler 3-dimensional models have identified that persistent Ca^2+^ waves can be generated through specific patterns of 3-dimensional Ca^2+^-wave propagation (so-called “ping waves”) (Thul et al., 2012), suggesting a need to consider the 3-dimensional structure of cardiomyocytes. Furthermore, although we increased the spatial resolution of our previously published model (Voigt et al., 2014) in order to simulate the banded pattern of RyR2 expression, the resolution of the current model (units of 1 μm^2^) was insufficient to simulate local Ca^2+^ dynamics around a single Ca^2+^-release unit (e.g., (sub)sparks). Detailed Ca^2+^-release unit models have been developed (Hake et al., 2012; Zahradnikova and Zahradnik, 2012; Walker et al., 2014) and could potentially be integrated in the present cell-level model in future studies, although this would significantly increase the computational complexity. Additionally, we acknowledge that our investigations into the effects of beta-adrenergic stimulation represent a highly simplified approach. Implementing a complete signaling pathway and its downstream effects on atrial electrophysiology, such as previously described for ventricular cardiomyocytes (Heijman et al., 2011) would be of interest, but was beyond the scope of the present study.

The resolution and quality of confocal imaging is limited by physical properties and quality of cell-isolation and antibody staining (particularly in rabbit samples). The use of super-resolution microscopy would be beneficial to obtain a more detailed overview of the 3-dimensional RyR2 distribution. In this case, dual staining of RyR2 and axial tubules should be performed to obtain information on both distributions in a single cardiomyocyte, which was not performed in the current study. Instead, a representative experimental axial tubule geometry from previously published work (Brandenburg et al., 2016) was used. Ideally, future experiments would be performed in human atrial cardiomyocytes to obtain a human-specific RyR2 distribution, rather than the rabbit atrial cardiomyocytes employed here, although their availability and cell-quality is generally much lower. Indeed, the experimental RyR2 distribution that formed the basis for the model with physiological RyR2 and LTCC distribution (Figure 10) was based on a limited number of rabbit atrial cardiomyocytes and may not be representative for diseased human atrial cardiomyocytes. Likewise, the current model requires stretching of the observed RyR2 expression pattern to match the 100x18 rectangular shape of the virtual cardiomyocyte. Finally, heterogeneous distributions of other Ca^2+^-handling proteins such as NCX1 or SERCA2a may also impact whole-cell Ca^2+^-handling and could be studied using the computational framework developed in the present study.

## Conclusions

We employed the perfect control and observability provided by computer models to overcome experimental challenges in the analysis of the subcellular determinants of cardiomyocyte Ca^2+^-handling. Our findings highlight the importance of atrial subcellular structures, especially RyR2 and LTCC distributions, in the genesis of SCaEs and DADs, which are well-known triggers of cardiac arrhythmias. Importantly, whole-cell Ca^2+^-handling properties are determined by non-linear interactions between heterogeneities in the properties (expression, phosphorylation) of both LTCC and RyR2, highlighting the need for detailed immunocytochemistry and functional studies to explain differences in whole-cell Ca^2+^-handling properties between conditions.

## Author contributions

HS, BvS, and JH conceived the study. PS, BvS, JH, MvZ, and GA performed and supervised the experiments. HS and JH performed the computational simulations and data analysis. HS and JH drafted the manuscript. All authors critically revised the manuscript and approved the final version.

### Conflict of interest statement

The authors declare that the research was conducted in the absence of any commercial or financial relationships that could be construed as a potential conflict of interest.

## Abbreviations

AF: atrial fibrillation
Ca^2+^: calcium
CICR: Ca^2+^-induced Ca^2+^-release
CRU: Ca^2+^ Release Unit
DAD: delayed after depolarization
IgG1: Immunoglobulin-G1
K^+^: potassium
LTCC: L-type Ca^2+^ channel
NA: Numerical Aperture
Na^+^: sodium
NCX1: Na^+^/Ca^2+^ exchanger type-1
RyR2: ryanodine receptor type-2
SCaE(s): spontaneous Ca^2+^-release event(s)
SR: sarcoplasmic reticulum
SERCA2a: SR Ca^2+^-ATPase type-2a.
