# Supplementary material for: The Subcellular Distribution of Ryanodine Receptors and L-Type Ca2+ Channels Modulates Ca2+-Transient Properties and Spontaneous Ca2+-Release Events in Atrial Cardiomyocytes
Source: Front Physiol. 2018 Aug 14;9:1108. doi: 10.3389/fphys.2018.01108 (PMC6107030; doi:10.3389/fphys.2018.01108)
Supplement: Supplementary file 1 [file Data_Sheet_1.PDF]

Supplemental Material

The Subcellular Distribution of Ryanodine Receptors and L-type Ca^{2+} Channels Modulates Ca^{2+}-transient Properties and Spontaneous Ca^{2+}-release Events in Atrial Cardiomyocytes

Henry Sutanto[a,#], Bart van Sloun[a,$,#], Patrick Schönleitner[b], Marc van Zandvoort[c], Gudrun Antoons[b], Jordi Heijman[a,*]

[a] Department of Cardiology, Maastricht University, Maastricht, The Netherlands

[b] Department of Physiology, Maastricht University, Maastricht, The Netherlands

[c] Department of Genetics & Cell Biology, Maastricht University, Maastricht, The Netherlands

[$] Present address: Maastricht Centre for Systems Biology (MaCSBio), Maastricht University, Maastricht, The Netherlands

[#]These authors contributed equally to this manuscript.

***** Corresponding author
(Jordi.Heijman@MaastrichtUniversity.nl)

Supplemental Methods

Animal model and cardiomyocyte isolation

New Zealand White rabbits (2.5-3.5 kg) were anesthetized with ketamine/xylazine via intramuscular injection. Inhalation anesthesia was maintained using mechanical ventilation with 0.5% isoflurane. For atrial cardiomyocyte isolation, hearts were quickly excised and placed in cold primary solution containing (in mM) NaCl 133, KCl 5, $MgCl_2 \cdot 6H_2O$ 2, KH_2PO_4 1.2, taurine 6, creatine 6, glucose 10, and HEPES, 10; pH 7.4, and were gently washed to clear the tissue from blood. Hearts were retrogradely perfused with primary solution via the aorta on a gravity-based Langendorff apparatus (80 cm H_2O) for 8 minutes to flush out remaining blood. Subsequently, hearts were perfused with primary solution supplemented with 15µM $CaCl_2$, 0.15 g/L Collagenase P (Roche 11213865), 0.40 g/L Collagenase B (Roche 11088815), 0.15 g/L Trypsin inhibitor (Roche 1019878), and 0.25 g/L Hyaluronidase (Sigma H-3506) for a total of 15 minutes until the atria softened. The left atrium was cut and transferred to a beaker containing 20 mL of storage solution (primary solution supplemented with 0.50 g of Bovine Serum Albumin and 15 µM of $CaCl_2$ per 100 ml). Atrial tissue was cut in smaller pieces and gently shaken to dissociate cells. The cell suspension was filtered using a nylon mesh (200 µm). Before plating the cells, Ca^{2+} concentration was increased step-wise (in mM: 0.05, 0.1, 0.2, 0.4, 0.8, 1) every 10 minutes up to a final concentration of 1 mM. All animal handling conformed with directive 2010/63/EU and experimental protocols were approved by the local ethical committee (DEC2014-112).

Immunocytochemistry

Atrial cardiomyocytes were seeded on laminin-coated coverslips. Cardiomyocytes were fixed in 2% (w/v) paraformaldehyde in phosphate-buffered saline (PBS, pH 7.4) for 20 minutes. After fixation, the cells were washed and then permeabilized with PBS containing 0.5% (v/v) Triton X-100. Cells were blocked with 10% (v/v) normal goat serum for 1 hour. Cardiomyocytes were incubated with a primary antibody raised against cardiac specific RyR2 (mouse monoclonal (C3-33), IgG1, Sigma-Aldrich®, MO, 1:50) for 2 hours at room temperature. After incubation with the primary antibody, the cells were washed three times with PBS, and incubated with a fluorophore-conjugated secondary antibody (Alexa® 488, goat anti-mouse, 1:100, Abcam). After the second incubation cells were washed three times with PBS. Coverslips were mounted with MOWIOL-based embedding medium containing 2.5% DABCO (Sigma Aldrich) as antifade.

Confocal microscopy

Images of the RyR2-stained atrial cardiomyocytes were acquired with a Leica TCS SP8 confocal microscope using a 63x objective (NA 1.40, oil immersion). The stained cardiomyocyte samples were transferred onto the motorized microscope stage. The RyR2-Alexa488 antibody complex was excited using a white-light laser at a wavelength of 488 nm. The fluorescence was detected using a HyD-1 detector at a wavelength of 420-520 nm with a smart gain of 80. Line averaging was set to 2 to reduce the image noise without compromising detail. Z-stacks were taken with a step size of 0.26 μm and a xy-resolution of 0.07 μm.

Image analysis

Image processing was performed to enable the integration into the human atrial cardiomyocyte model. The raw z-stacks were deconvolved using Huygens Professional (SVI, Netherlands) to correct for microscope point-spread function (**Supplemental Figure 1A**). A theoretical point spread function was generated and used to determine the performance of the objective and imaging system. Deconvolution was initiated using the fast-classical maximum likelihood estimation algorithm with a signal to noise ratio of 20 and a maximum of 40 iterations. The deconvolved z-stack was loaded into imageJ. A single slice was selected from the z-stack and rotated to obtain a horizontal alignment of the RyR2s. A region of interest was selected and imported into MATLAB 2017b (**Supplemental Figure 1B**). The resulting image was thresholded, i.e., all pixel values below 500 were set to zero. Next, the image was divided using a grid of ~1 μm^2 units (**Supplemental Figure 1C**), in which the mean intensity for every square in the grid was calculated and stored into a mean intensity matrix. The edges of the mean intensity matrix were detected using a threshold for the RyR2 expression, and used to stretch the matrix into 18 transversal domains of 1 μm. The resulting 32x18 RyR2 expression matrix was replicated ~3 times to obtain a 100x18 RyR2 expression matrix reflecting a RyR2 distribution suitable for simulation using the human atrial computational model.

Computational modeling

The simulations in this manuscript were based on a previously published model of the human atrial cardiomyocyte (1) coded in C++ and interfaced to Octave (2). In this model the virtual cardiomyocyte has been divided into 50 longitudinal segments of ~2 μm and 18 transverse

subcellular Ca^{2+} domains of ~1 μm, reflecting a 100 by 18 μm cardiomyocyte. In each unit, changes in the local concentration of Ca^{2+} in the SR, cytosol and SR Ca^{2+}-release space, representing the immediate vicinity around the RyR2 channels, due to local ion fluxes and diffusion of Ca^{2+} between units are simulated over time. In the published model the RyR2 distribution was identical in every unit. In the present work, the model was extended to enable simulation with heterogeneous distributions of RyR2.

An algorithm was written to create RyR2 distributions with varying degrees of heterogeneity. The RyR2 distribution was scaled using a Gaussian distribution with mean 1.0 and variable standard deviation (σ). The total number of RyR2 was kept the same for varying RyR2 distributions, unless indicated otherwise. Changes in total RyR2 expression were simulated by scaling both the number of stochastic RyR2 (N_{RyR} parameter) and the total RyR2 conductance ($p[11]_{RyR2}$ in the description of the original model (1)) in order to maintain fixed single-channel conductance. In addition, RyR2 parameters could be changed in every Ca^{2+} unit to simulate local changes in RyR2 open probability, e.g., due to hyperphosphorylation.

Experimental data showed that the RyR2 appear in a banded pattern throughout the cardiomyocyte, known as the z-bands, with an inter-band distance of approximately 2 μm. As such, the spatial resolution of the human atrial cardiomyocyte model was increased to 100 longitudinal segments of ~1 μm and 18 transverse subcellular Ca^{2+} domains of ~1 μm to be able to simulate the banded RyR2 pattern. The algorithm used to create the RyR2 distributions was extended to create RyR2 distributions without inter-band RyR2 expression (i.e., the RyR2 expression of the inter-band was set to zero, with the exception of the first and last domain). Recent work has shown that under diseased conditions more RyR2 clusters are expressed between the z-bands. The algorithm was therefore further extended to incorporate inter-band RyR2 expression. A ten percent chance of RyR2 expression in each domain of the segment located between RyR2 bands was incorporated into the algorithm, again excluding the first and last domain.

Similar to the ability to simulate arbitrary RyR2-expression patterns, the model was extended to simulate arbitrary L-type Ca^{2+}-channel (LTCC) expression. Various numbers and locations of LTCC distributions were incorporated in the 100-segment model (independently from the exact RyR2 distribution) to represent axial tubules in the atrial cardiomyocyte. Non-parallel axial tubules were incorporated in segment 75-94, domain 5 and segment 5-24, domain 13.

The parallel axial tubules were implemented by incorporating LTCCs in segment 5-24, domain 5 and segment 5-24, domain 13. Furthermore, a central axial tubule was represented by LTCC in segment 5-24, domain 9. For the experimentally observed axial tubules, we implemented a pattern previously published by Brandenburg et al (3). Simulations were performed and transversal wave line scans were recorded. The shapes of transversal wave line scans were compared amongst the groups, local and global time-to-peak and global Ca^{2+} amplitude were measured. Local hyperphosphorylation of RyR2 adjacent to the axial tubules, as observed by Brandenburg et al. (3) was simulated by increasing RyR2 open probability at the site of axial tubules. Increased RyR2 open probability was simulated using the changes in parameters employed in the original model for patients with paroxysmal AF: $P[0]_{RyR} = 0.2 \rightarrow 0.4$ (+100%); $P[1]_{RyR} = 0.22 \rightarrow 0.24$ (+9%); $P[5]_{RyR} = 0.0035 \rightarrow 0.0105$ (+200%) (1).

An algorithm was written to detect spontaneous sarcoplasmic reticulum (SR) Ca^{2+}-release events (SCaEs) and determine their properties. In particular, after the initiation of a SCaE in a given unit (first local maximum in Ca^{2+} over time), the neighboring units were examined. If neighboring units had a local maximum in intracellular Ca^{2+} within 100 milliseconds, these local maxima were considered to be part of the same propagating wave front and were used to calculate the wave size (as fraction of the total cell size). The distance between the initiating unit and the last unit of a wave was calculated and divided by the time since the initiation of the SCaE to obtain the (average) longitudinal and transversal velocity of the entire SCaE. The output of the algorithm was manually validated for 50- and 100-segment simulations with uniform RyR2 distribution.

**Parameter**	**Published model (1)**	**50-segment model**	**100-segment model**	**Exp RyR2 + axial tubules + hyperphos. RyR**
$\tau_{diff,SRS \leftrightarrow cyt}$ **(ms)**	**12**	**12**	**10**	**10**
$\tau_{diff,seg,SRS}$ **(ms)**	**0.3**	**0.3**	**0.22**	**0.10**
$\tau_{diff,dom,SRS}$ **(ms)**	**0.125**	**0.125**	**0.16**	**0.10**
$\tau_{diff,seg,cyt}$ **(ms)**	**0.6**	**0.6**	**0.6**	**0.6**
$\tau_{diff,dom,cyt}$ **(ms)**	**0.6**	**0.6**	**0.6**	**0.6**
$\tau_{diff,seg,SR}$ **(ms)**	**15**	**15**	**5**	**5**
$\tau_{diff,dom,SR}$ **(ms)**	**15**	**15**	**5**	**5**
N_{RyRs}	**594,000**	**514,800**	**2,772,000**	**79,200,000**
**SERCA2a (Kmr)**	**1.00**	**1.00**	**1.25**	**1.25**
I_{CaL} **parameters**		**As in (4):**	**As in (4):**	**As in (1) with adjusted** P_1**:**
	P_1 = 9.45	P_1 = 9.45	P_1 = 9.45	P_1 = 8.50
	P_2 = 49.1	P_2 = 65.0	P_2 = 65.0	P_2 = 49.1
	P_3 = 10.349	P_3 = 6.000	P_3 = 6.00	P_3 = 10.349
	P_4 = 26.553	P_4 = 12.00	P_4 = 12.00	P_4 = 26.553
	P_5 = 17.5	P_5 = 27.5	P_5 = 27.5	P_5 = 17.5
	P_6 = 3.0	P_6 = 5.0	P_6 = 5.0	P_6 = 3.0
	P_7 = 13.825	P_7 = 43.825	P_7 = 43.825	P_7 = 13.825
	P_8 = 6.3836	P_8 = 45.00	P_8 = 45.00	P_8 = 6.3836
	P_9 = 14.9186	P_9 = 5.000	P_9 = 5.000	P_9 = 14.9186
	P_{10} = 1.100	P_{10} = 5.000	P_{10} = 5.000	P_{10} = 1.100

Supplemental Table 1. Parameters changes applied to the published model (1) with 50 segments to fit the previously published experimental data. Subsequently, slight modifications of the parameters, including the number of RyR (N_{RyRs}), Kmr of SERCA and time constant (τ) SRS, were made to the 100-segment models to obtain similar physiological Ca^{2+}-handling properties.

For the L-type Ca^{2+} channel, the 8-state Markov model was used as previously described (1, 4). Parameters of this Markov model are as follows:

$$ACT_\tau = 0.59 + 0.8 \cdot \frac{\exp\left(0.052 \cdot (V_m + 13)\right)}{1 + \exp\left(0.132 \cdot (V_m + 13)\right)}$$

$$ACT_\infty = \frac{1}{\left(1 + \exp\left(-\frac{V_m - 13.56}{P_1}\right)\right) \cdot \left(1 + \exp\left(-\frac{V_m + 25}{5}\right)\right)}$$

$$\alpha_{Ca,L} = \frac{ACT_\infty}{ACT_\tau}, \qquad \beta_{Ca,L} = \frac{1 - ACT_\infty}{ACT_\tau}$$

$$I_{V,\infty} = \frac{1}{1.2474} \cdot \left(0.2474 + \frac{1}{1 + \exp\left((V_m + P_5)/P_6\right)}\right)$$

$$Is_{V,\infty} = \frac{1}{1.001} \cdot \left(0.001 + \frac{1}{1 + \exp\left((V_m + P_5)/P_6\right)}\right)$$

$$I_{V,\tau} = \frac{1}{\frac{1}{70.0 \cdot \left(1 + \exp\left((V_m + P_2)/P_3\right)\right)} + \frac{1}{P_4 \cdot \left(1 + \exp(-(V_m + 0.213)/10.807)\right)}}$$

$$Is_{Ca}^{s,m} = P_7 - \frac{P_8}{1 + \left(\frac{3.500 \cdot 10^{-4}}{[Ca^{2+}]_{obs}^{s,m}}\right)^4}$$

$$Is_{V,\tau}^{s,m} = \frac{1}{\frac{1}{70.0 \cdot \left(1 + \exp\left((V_m + 49.10)/10.349\right)\right)} + \frac{1}{Is_{Ca}^{s,m} = \left(1 + \exp(-(V_m + 0.213)/10.807)\right)}}$$

$$x_{Ca,L} = \frac{I_{V,\infty}}{I_{V,\tau}}, \qquad y_{Ca,L} = \frac{1 - I_{V,\infty}}{I_{V,\tau}}$$

$$xs_{Ca,L}^{s,m} = \frac{Is_{V,\infty}}{Is_{V,\tau}}, \qquad ys_{Ca,L}^{s,m} = \frac{1 - Is_{V,\infty}}{Is_{V,\tau}}$$

$$\delta_{Ca,L}^{s,m} = \frac{P_9}{1 + \left(\frac{P_{10} \cdot 10^{-3}}{[Ca^{2+}]_{obs}^{s,m}}\right)^4}, \qquad \theta_{Ca,L} = 1$$

$$\theta I_{Ca,L} = 1.0 \cdot 10^{-6}, \qquad \delta I_{Ca,L}^{s,m} = \theta I_{Ca,L} \cdot \frac{x_{Ca,L} \cdot ys_{Ca,L}^{s,m} \cdot \delta_{Ca,L}^{s,m}}{y_{Ca,L} \cdot xs_{Ca,L}^{s,m} \cdot \theta_{Ca,L}}$$

$$P_{Ca,L} = 1.7 \cdot 10^{-4}\ \frac{cm}{s}, \qquad \gamma_{Ca,i} = 1.0, \qquad \gamma_{Ca,o} = 0.341$$

$$\bar{I}_{Ca,L}^{s,m} = P_{Ca,L} \cdot (z_{Ca})^2 \cdot \frac{V_m \cdot F^2}{R \cdot T} \cdot \frac{\gamma_{Ca,i} \cdot [Ca^{2+}]_{obs}^{s,m} \cdot \exp\left(z_{Ca} \cdot V_m \cdot \frac{F}{R \cdot T}\right) - \gamma_{Ca,o} \cdot [Ca^{2+}]_o}{\exp\left(z_{Ca} \cdot V_m \cdot \frac{F}{R \cdot T}\right) - 1}$$

$$I_{Ca,L}^{s,m} = \bar{I}_{Ca,L}^{s,m} \cdot \left(O_{Ca,L}^{s,m} + Os_{Ca,L}^{s,m}\right)$$

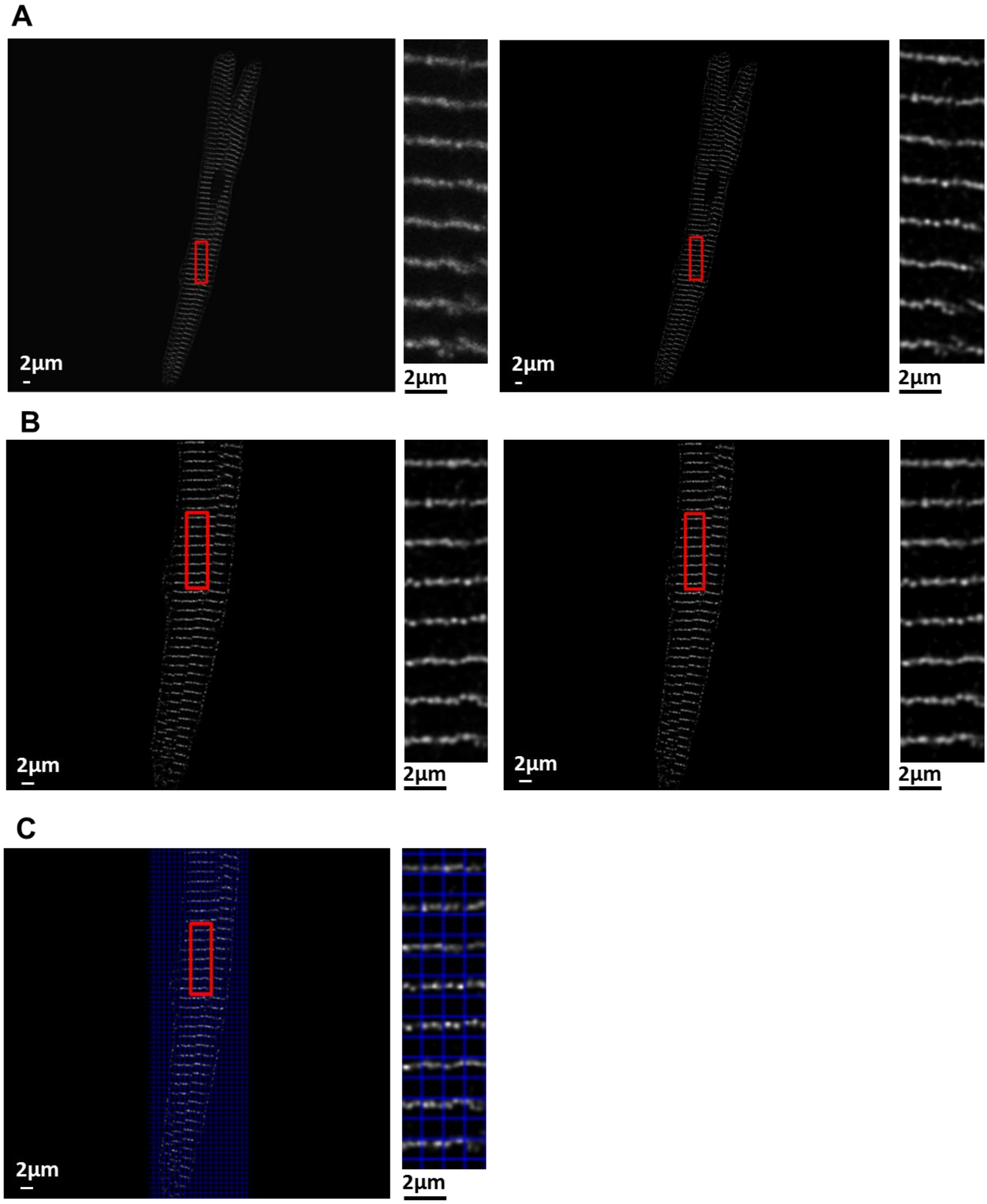

Supplemental Figure 1. Image processing workflow. A) Single slice of the RyR2 z-stack in a rabbit atrial cardiomyocyte stained with Alexa® 488 antibodies, before (left) and after deconvolution (right). B) Rotated region of interest before (left) and after thresholding (right). C) Alignment with a regular grid of ~1 µm^2 units, which was subsequently employed to determine the average RyR2 expression level. Smaller panels show region indicated in the red box at a higher magnification.

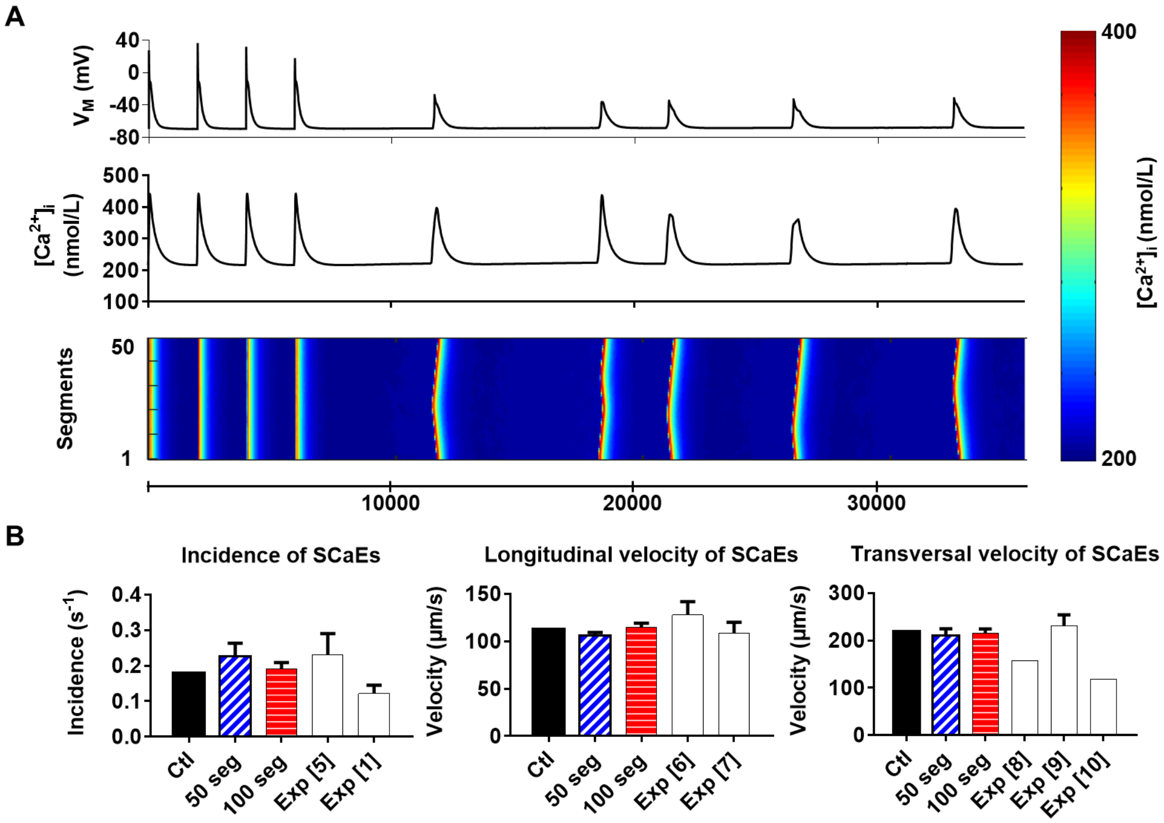

Supplemental Figure 2. Validation of spontaneous sarcoplasmic reticulum Ca^{2+}-release events (SCaEs) in the published (Ctl), adapted 50 segment (50 seg) and 100 segment (100 seg) models. A) Membrane potential (V_M), Ca^{2+} transient and longitudinal line scan of three triggered Ca^{2+} transients and 5 SCaEs during follow-up in the 50 seg model. B) Validation of SCaE incidence, as well as velocity of Ca^{2+} waves in longitudinal and transversal directions for the three model versions (based on n=6 simulations each) compared to experimental data (1, 5-10). Reference numbers are indicated in square brackets.

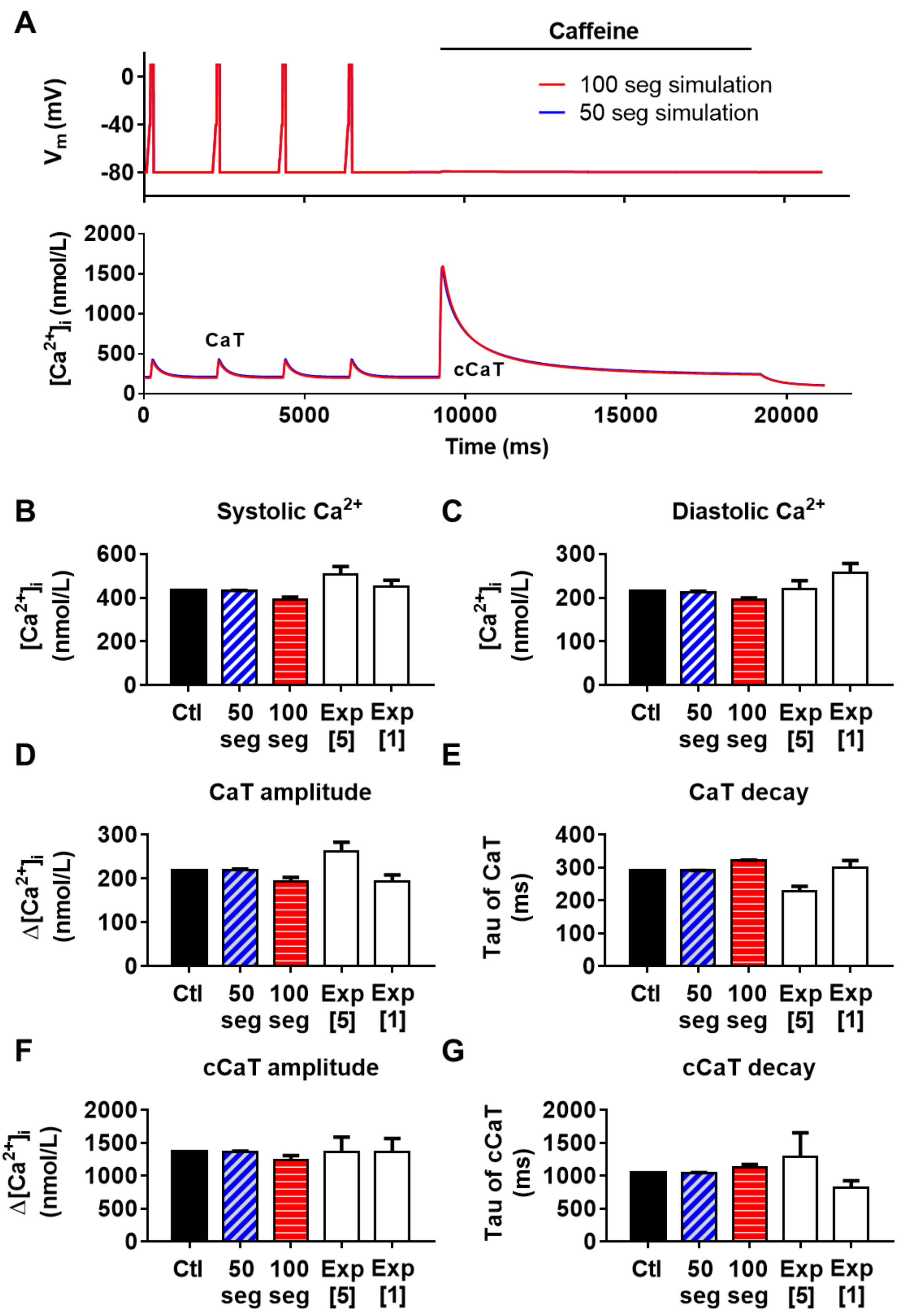

Supplemental Figure 3. Validation of Ca^{2+}-handling properties of the models. A) Membrane potential (V_M) and intracellular Ca^{2+} showing protocol used during simulation: Four triggered waves were shown prior to the simulated application of caffeine to induce Ca^{2+} release from the sarcoplasmic reticulum. B-G) Comparison of the Ca^{2+}-handling properties of control, 50-segment and 100-segment models to experimental data (1, 5). Reference numbers are indicated in square brackets.

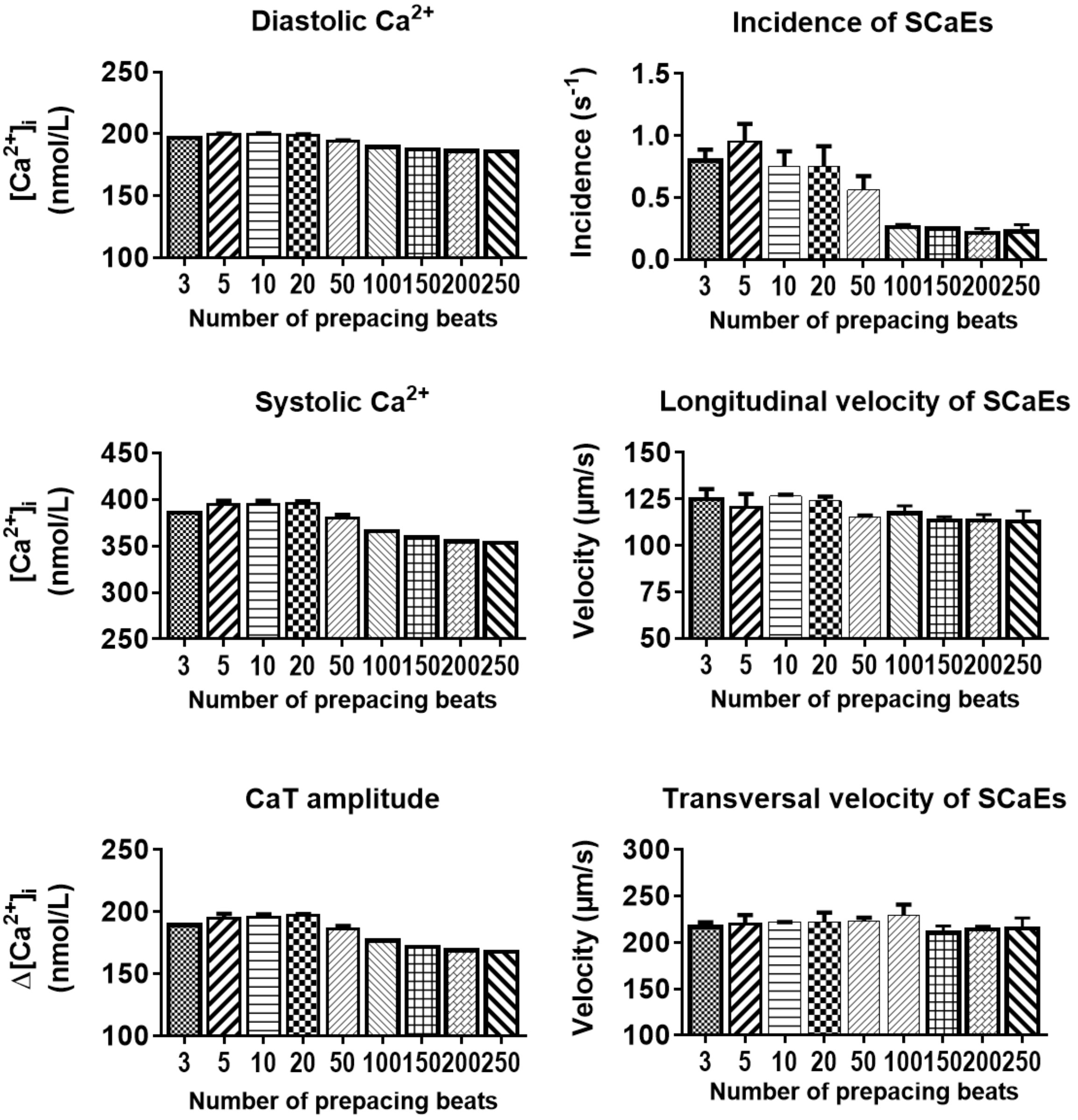

Supplemental Figure 4. Analysis of Ca^{2+}-handling properties for different prepacing durations in the 100-segment model. Simulations were run for various numbers of prepacing beats to find a value that represents a quasi-steady state condition. Ca^{2+}-handling parameters of triggered Ca^{2+} transients (diastolic levels, systolic levels and Ca^{2+}-transient amplitude; left panels) and SCaEs (incidence, longitudinal and transversal velocity; right panels) were documented. After prepacing for 200-250 beats, Ca^{2+}-handling parameters reach an acceptable steady state. Accordingly, results in the main manuscript for the 100-segment model are shown after 250 beats prepacing.

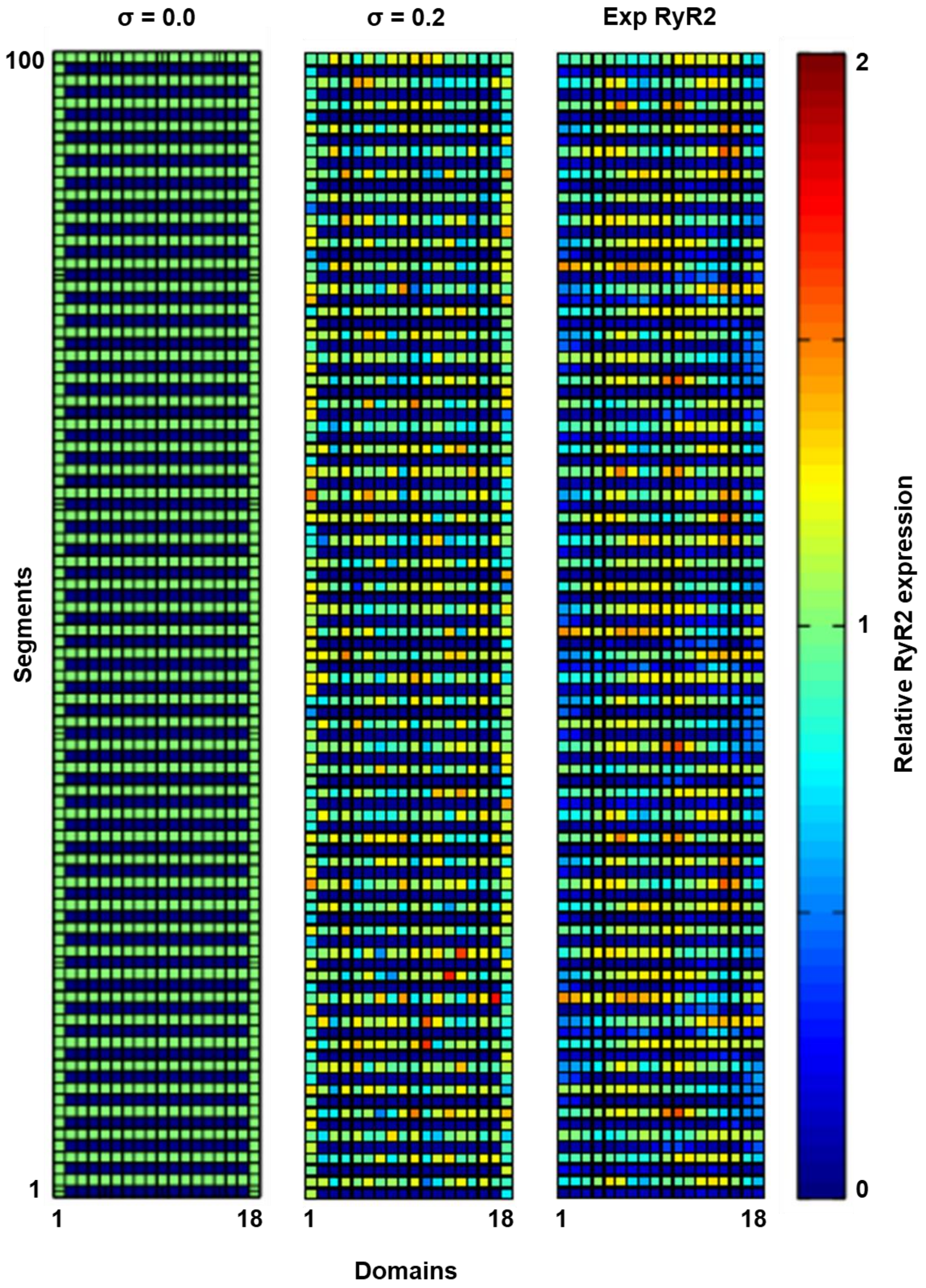

Supplemental Figure 5. Comparison of RyR2 distribution in 100-segment simulations with σ = 0.0, σ = 0.2, and experimental RyR2 distribution. Simulation of experimental RyR2 distribution displayed banded patterns with several areas with local high RyR2 expression.

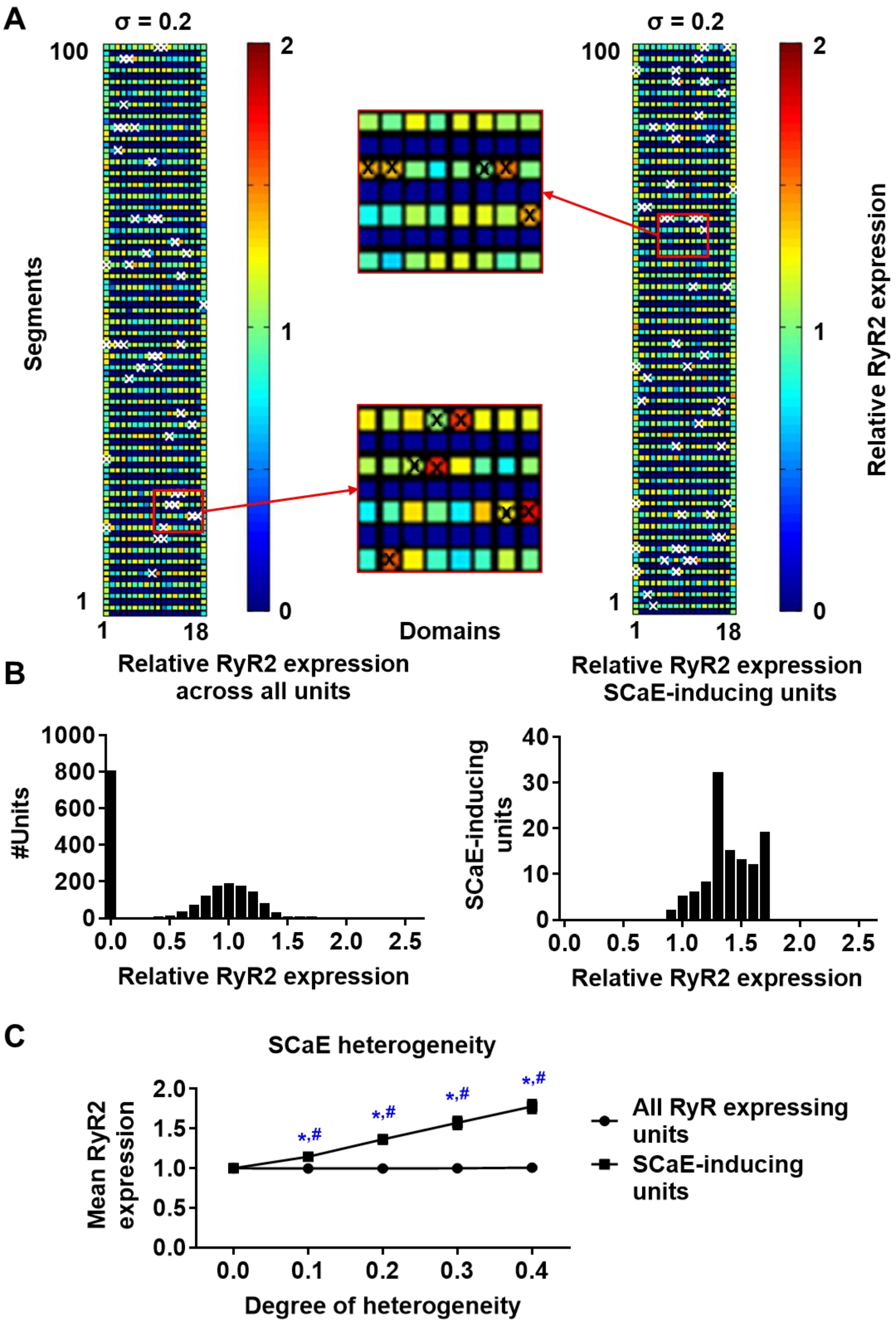

Supplemental Figure 6. Origins of spontaneous Ca^{2+}-release events (SCaEs). A) Two representative examples of 100x18 matrices with heterogeneous RyR2 distribution (σ = 0.2). Red colors indicate high local RyR2 expression and blue colors low local RyR2 expression. The origins of individual Ca^{2+} waves are marked with crosses. Insets depict enlarged portions of the RyR2 distribution. B) Histograms of relative RyR2 expression in all units (left) and

units which were the origin of a SCaE (SCaE-inducing units). C) Mean relative RyR2 expression in SCaE-inducing units (squares) and all units (circles, 1.0 by definition) for different degrees of RyR2 heterogeneities. Consistent with the 50-segments simulations (**Figure 2**), the spontaneous waves originated from units with local high RyR2 expression. * indicates $P<0.05$ vs. heterogeneity 0.0; # indicates $P<0.05$ between mean relative RyR2 expression in SCaE-inducing units and all units; n=6 per condition.

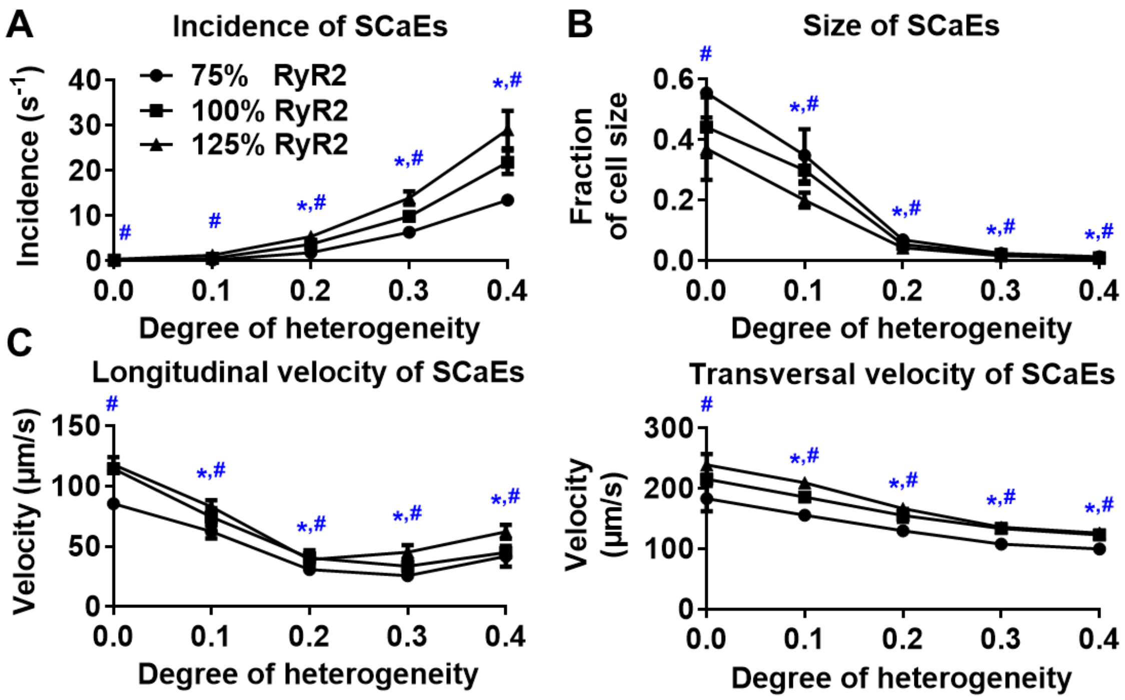

Supplemental Figure 7. Comparison of spontaneous Ca^{2+}-wave (SCaE) properties for various degrees of RyR2 heterogeneity and total expression in the 100-segment model. A-C) SCaE incidence (A) and size (B), as well as longitudinal and transversal velocity of Ca^{2+} waves (C) as a function of RyR2 heterogeneity σ for different levels of total RyR2 expression (75% of control: circles; 100% of control: squares; 125% of control: triangles). In line with the 50-segment simulations, increasing the total RyR2 expression also increased the incidence and lowered the size of the wave. * indicates $P<0.05$ vs. the group with heterogeneity 0.0; # indicates significant difference among the three levels of RyR2 expression; n=6 per condition.

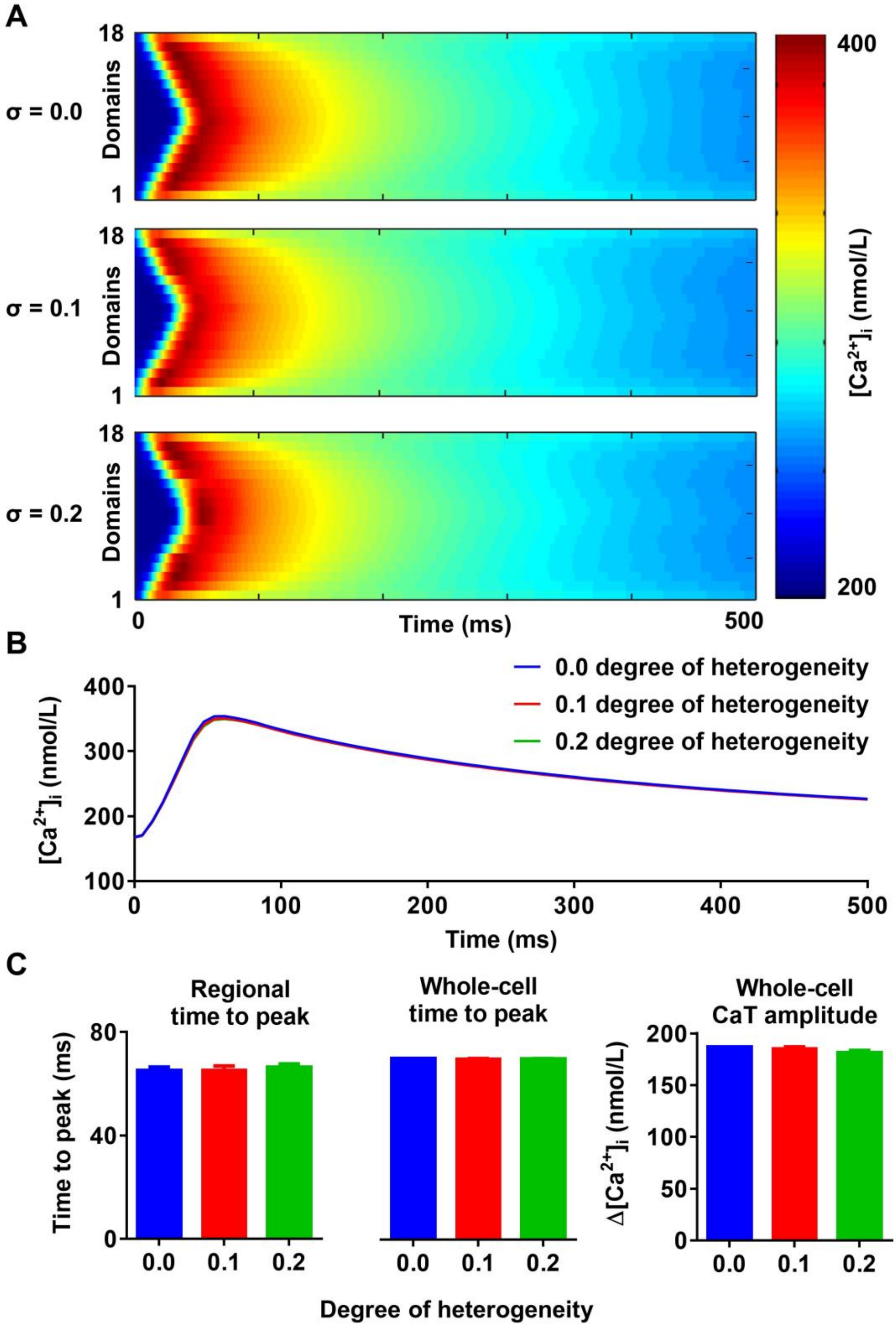

Supplemental Figure 8. Effect of RyR2 heterogeneity on systolic Ca^{2+}-wave propagation in the absence of SCaEs. Comparison of the transversal Ca^{2+} waves (A) and whole-cell Ca^{2+} transient (B) during 0.5 Hz pacing for three different degrees of heterogeneity. As expected, the "V" shape pattern of atrial Ca^{2+}-wave propagation can be observed, reflecting the lack of transversal tubules. C) Quantification of time to peak and Ca^{2+}-transient amplitude (right) for the indicated line scan ("regional") or for the whole-cell Ca^{2+} transient for three levels of RyR2 heterogeneity. RyR2 heterogeneity did not affect the characteristics of systolic Ca^{2+}-wave propagation.

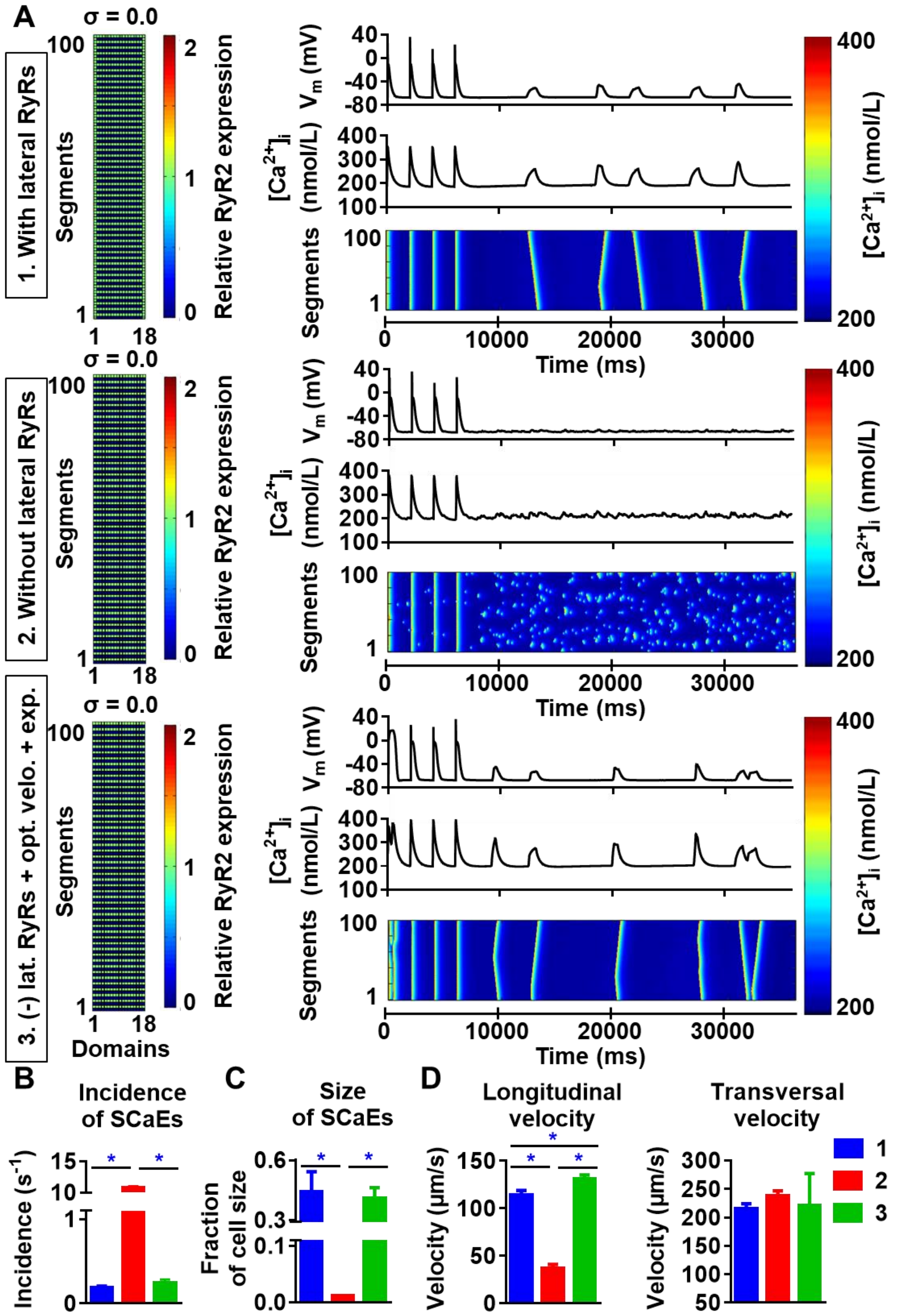

Supplemental Figure 9. The importance of lateral RyR2s in the propagation of SCaEs. A) Membrane potential (V_M), intracellular Ca^{2+} and longitudinal line scan (top to bottom) for the baseline model with lateral RyR2 (A.1), the model without lateral RyR2s (A.2), and the model without lateral RyR2 after the adjustment of total RyR2 expression and correction of longitudinal velocity (A.3). B-D, incidence (B), size (C), as well as longitudinal and transversal velocity of SCaEs for the three model conditions. Absence of lateral RyR2 resulted in more and smaller waves compared to baseline, presumably due to relocation and redistribution of RyR2. After correction of total RyR2 expression and longitudinal velocity, the incidence and size of SCaEs recovered. * indicates P<0.05 between groups; n=6 per condition.

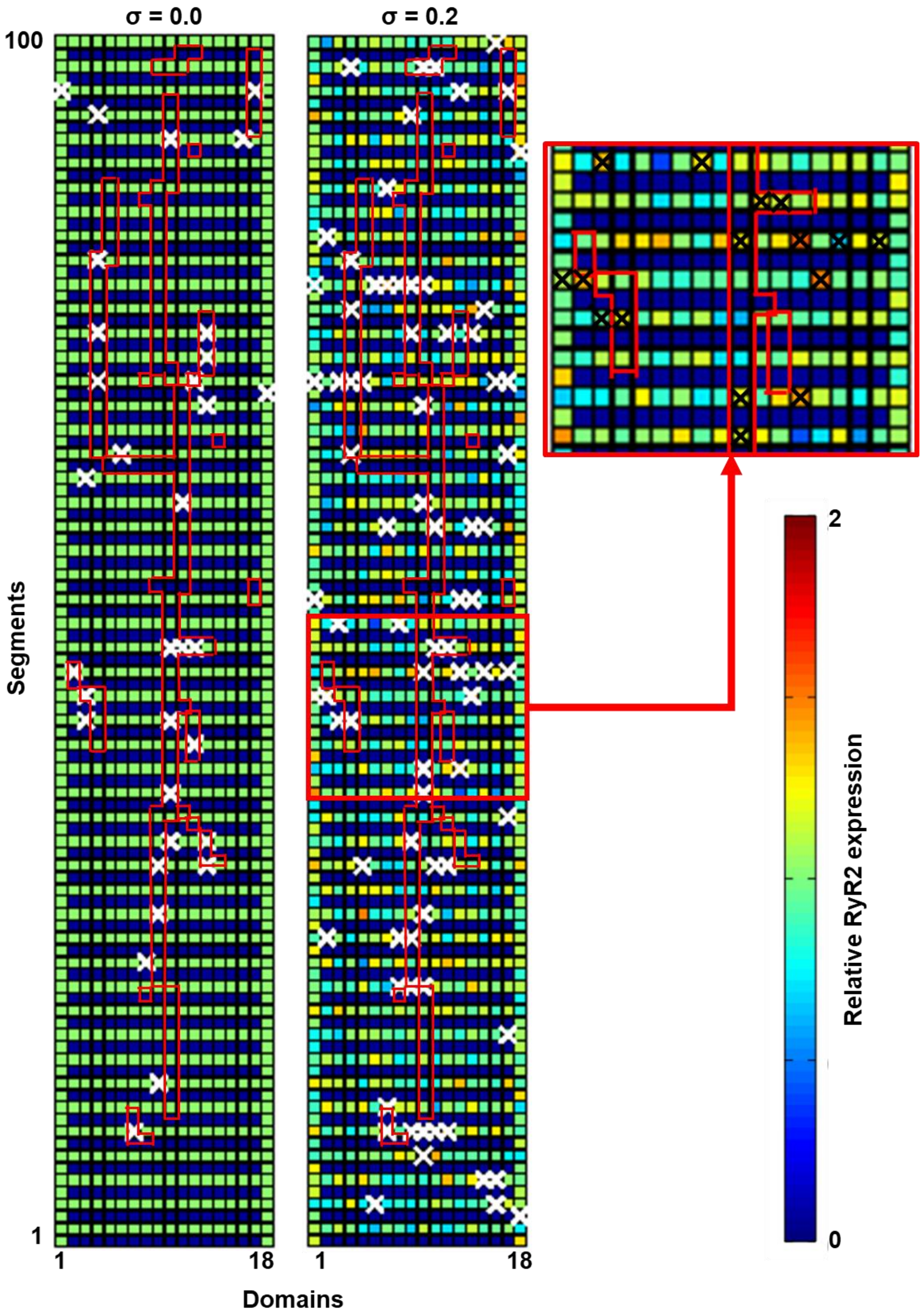

Supplemental Figure 10. Distribution of RyR2 expression and location of axial tubules (red lines) in relation to the origin of SCaEs (white crosses) for simulations with uniform (σ=0.0) or heterogeneous (σ=0.2) RyR2 expression. SCaEs originated primarily from RyR2 clusters adjacent to the axial tubules or (in the case of heterogeneous RyR2 expression) clusters with high local RyR2 expression (e.g., see inset in which black crosses indicate the origin of SCaEs).

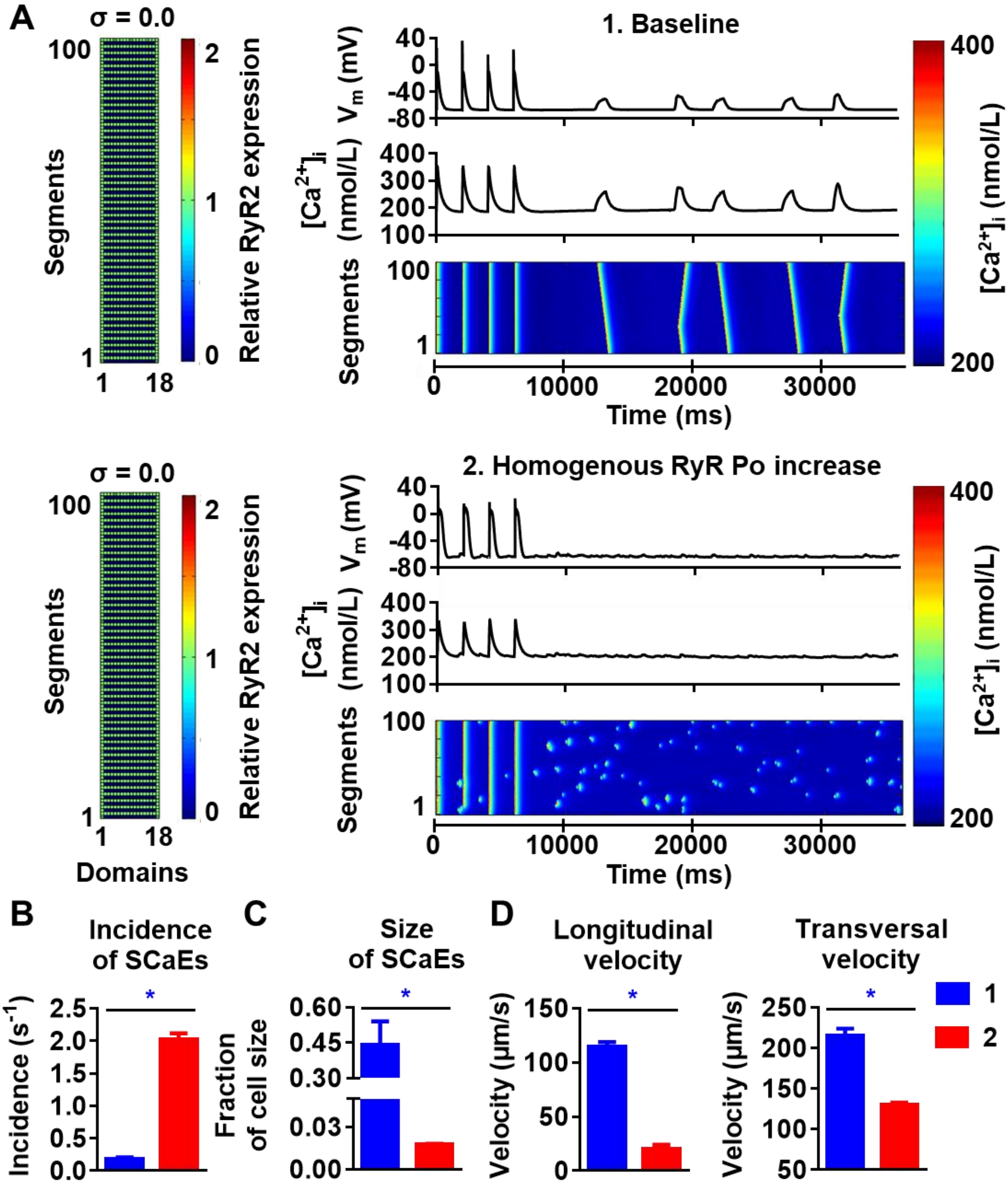

Supplemental Figure 11. Effects of homogenous increase in RyR open probability. A) Membrane potential (V_M), intracellular Ca^{2+} and longitudinal line scan (top to bottom) for the baseline model (A.1) and the model in which RyR2 open probability was increased by 100%, by altering RyR2 parameters as follows: $P[0]_{RyR}$ = 0.2 → 0.4 (+100%); $P[1]_{RyR}$ = 0.22 → 0.24 (+9%); $P[5]_{RyR}$ = 0.0035 → 0.00805 (+130%). B-D) incidence, size and velocity of SCaEs in both models. * indicates $P<0.05$ between both groups (n=6 per condition).

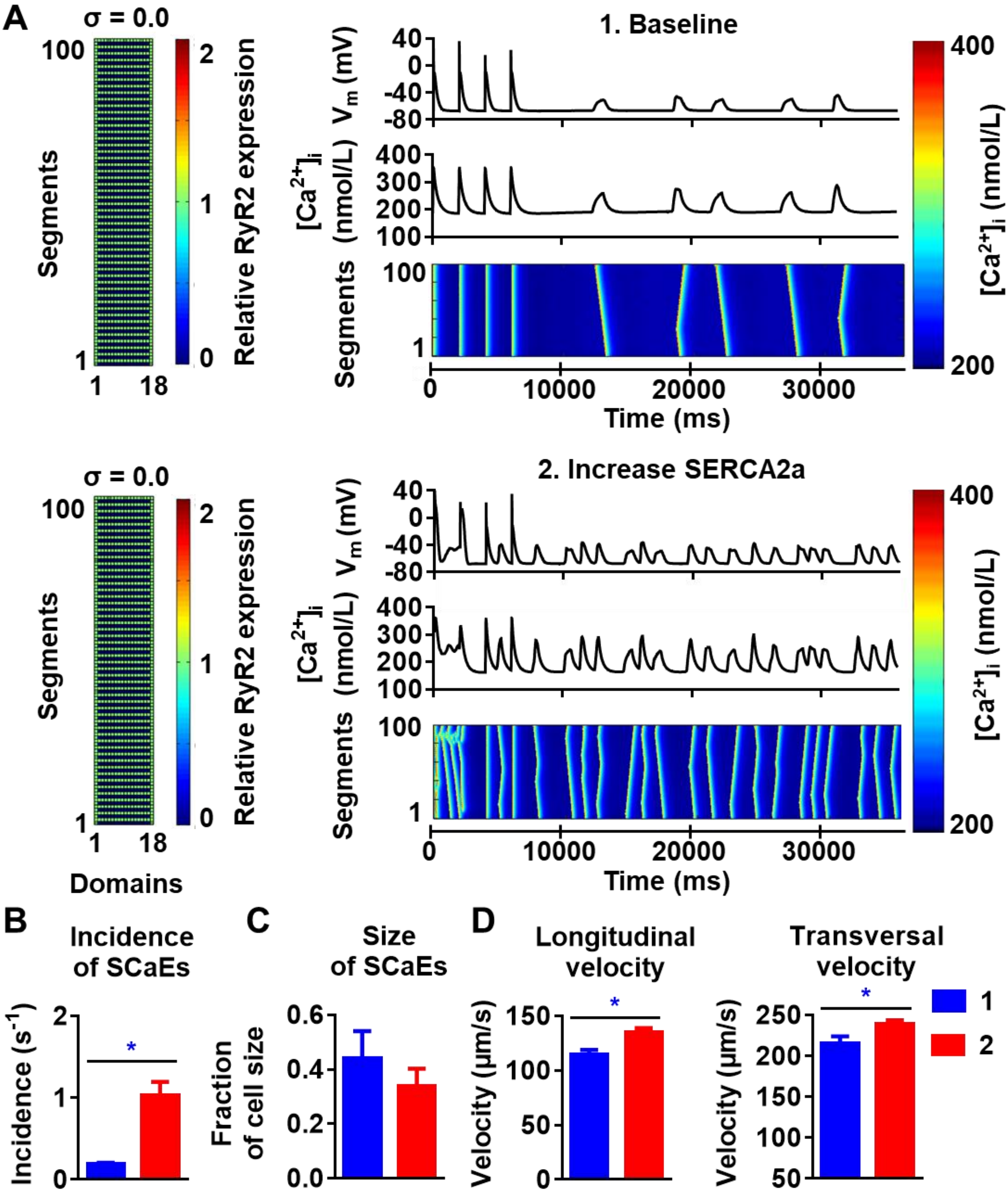

Supplemental Figure 12. Increase of SERCA2a function and the propensity of SCaEs. A) Membrane potential (V_M), intracellular Ca^{2+} and longitudinal line scan (top to bottom) for the baseline model (A.1) and the model with increased SERCA2a activity as achieved by reduction of Kmf value by 25% (A.2). B-D) incidence, size and velocity of SCaEs in both models. * indicates P<0.05 between both groups (n=6 per condition). The increase in SERCA2a activity resulted in an increased incidence of SCaEs with no significant change in size but significant increase in longitudinal and transversal velocities.

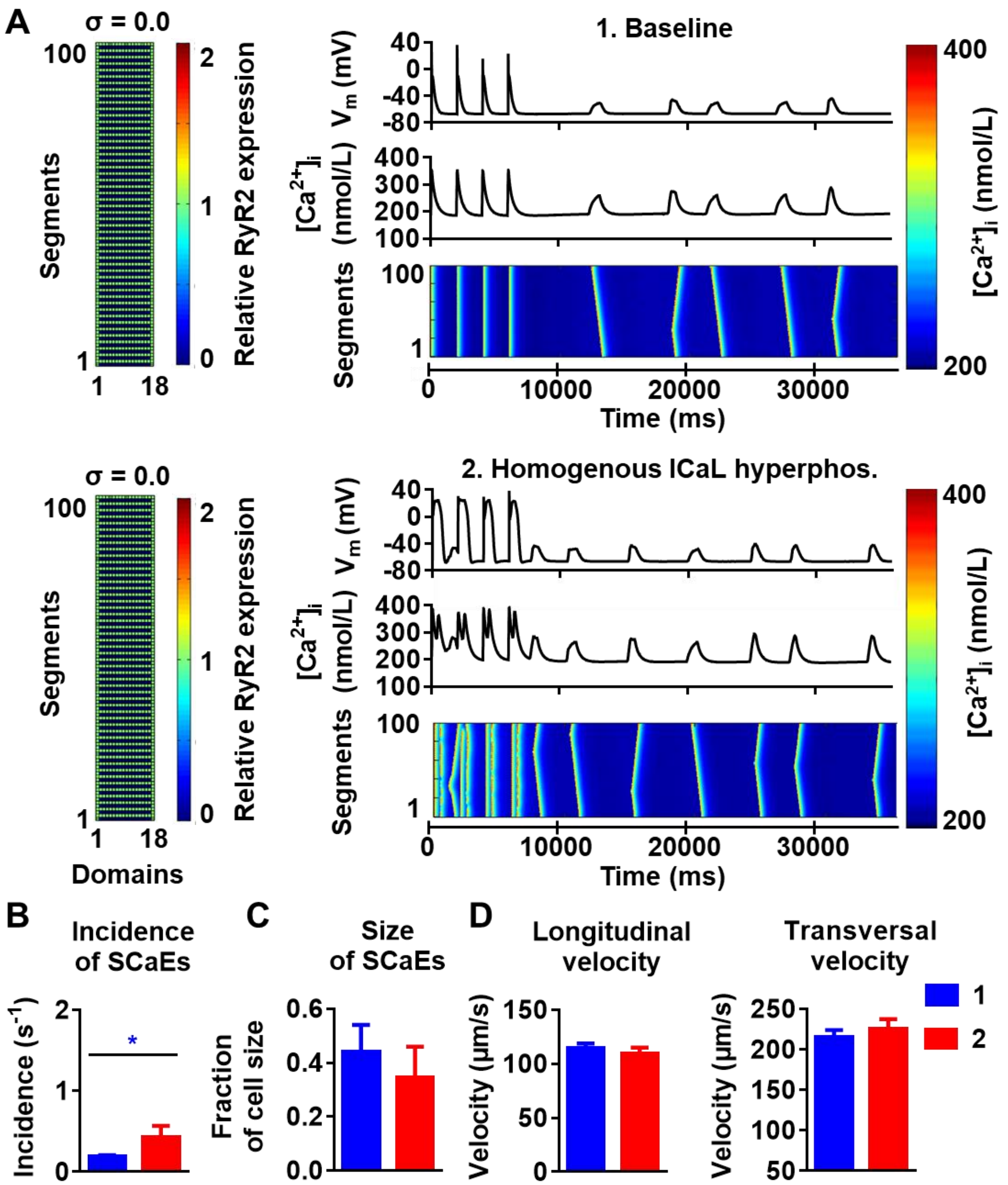

Supplemental Figure 13. Effects of homogenous LTCC/I_{CaL} hyperphosphorylation on the behavior of SCaEs. A) Membrane potential (V_M), intracellular Ca^{2+} and longitudinal line scan (top to bottom) for the baseline model (A.1) and the model with doubled maximum conductance of LTCC (from $1.7x10^{-4}$ as reported before to $3.4x10^{-4}$) (A.2). B-D) incidence, size and velocity of SCaEs in both models. * indicates $P<0.05$ between both groups (n=6 per condition). The increase in I_{CaL} resulted in increased SCaE incidence without any significant change in other SCaEs parameters.

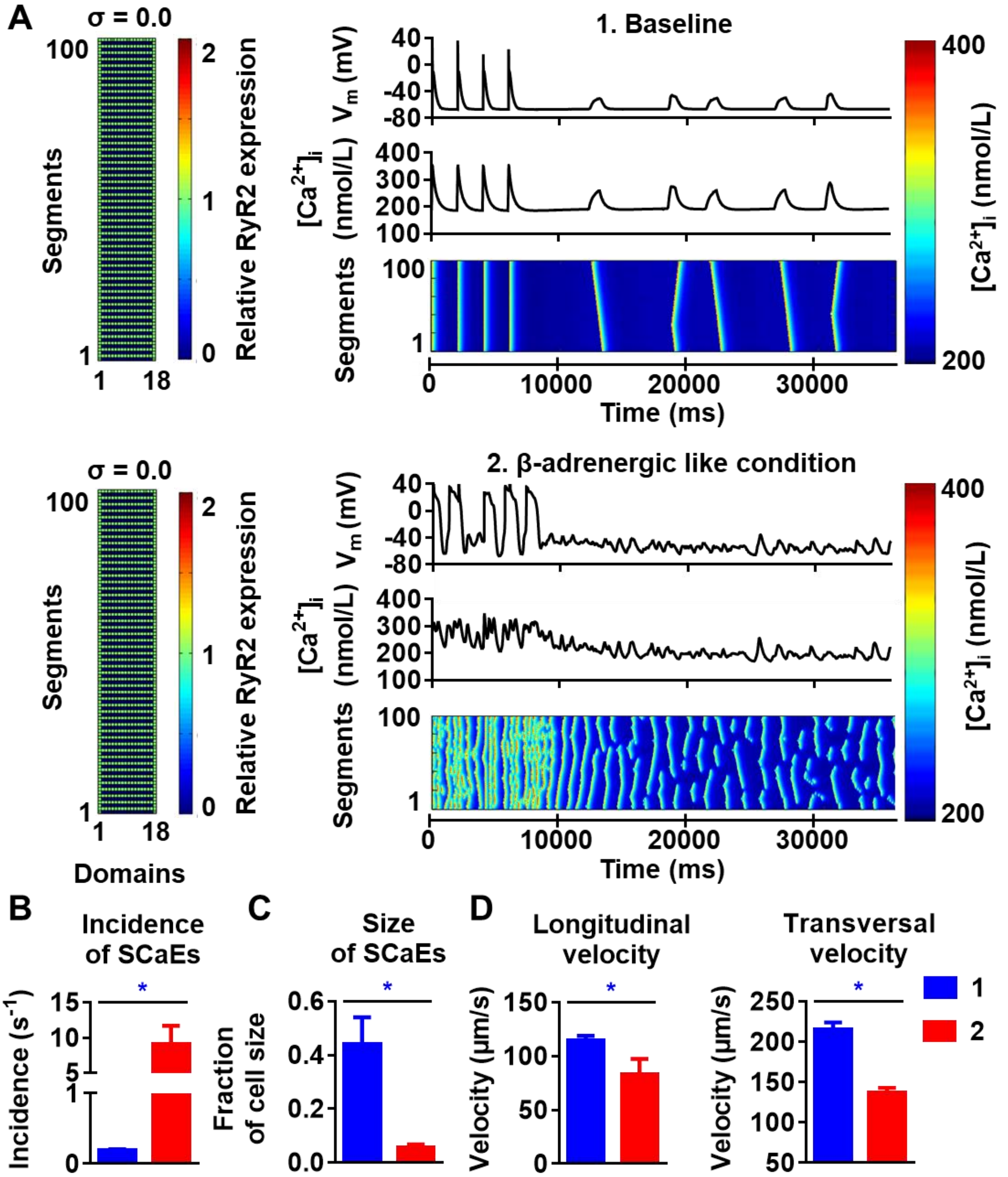

Supplemental Figure 14. The effect of simulated beta-adrenergic stimulation on the behavior of SCaEs. A) Membrane potential (V_M), intracellular Ca^{2+} and longitudinal line scan (top to bottom) for the baseline model (A.1) and the model with increased RyR2 open probability, increased SERCA2a activity and increased I_{CaL} (A.2.) B-D) incidence, size and velocity of SCaEs in both models. * indicates P<0.05 between both groups (n=6 per condition). The simulated beta-adrenergic stimulation markedly increased the incidence of SCaEs and reduces the size of SCaEs, longitudinal and transversal velocities of the spontaneous waves.

References

1. Voigt N, Heijman J, Wang Q, Chiang DY, Li N, Karck M, et al. Cellular and molecular mechanisms of atrial arrhythmogenesis in patients with paroxysmal atrial fibrillation. *Circulation* (2014) 129(2):145-56. doi: 10.1161/CIRCULATIONAHA.113.006641. PubMed PMID: 24249718.

2. Eaton J BS, Hauber R, Wehbring R. GNU Octave version 4.0.0 manual: a high-level interactive language for numerical computations (2015). Available from: http://www.gnu.org/software/octave/doc/interpreter/.

3. Brandenburg S, Kohl T, Williams GS, Gusev K, Wagner E, Rog-Zielinska EA, et al. Axial tubule junctions control rapid calcium signaling in atria. *J Clin Invest* (2016) 126(10):3999-4015. doi: 10.1172/JCI88241. PubMed PMID: 27643434.

4. Heijman J, Erfanian Abdoust P, Voigt N, Nattel S, Dobrev D. Computational models of atrial cellular electrophysiology and calcium handling, and their role in atrial fibrillation. *J Physiol* (2016) 594(3):537-53. doi: 10.1113/JP271404. PubMed PMID: 26582329.

5. Voigt N, Li N, Wang Q, Wang W, Trafford AW, Abu-Taha I, et al. Enhanced sarcoplasmic reticulum Ca^{2+} leak and increased Na^{+}-Ca^{2+} exchanger function underlie delayed afterdepolarizations in patients with chronic atrial fibrillation. *Circulation* (2012) 125(17):2059-70. doi: 10.1161/CIRCULATIONAHA.111.067306. PubMed PMID: 22456474.

6. Loughrey CM, Smith GL, MacEachern KE. Comparison of Ca^{2+} release and uptake characteristics of the sarcoplasmic reticulum in isolated horse and rabbit cardiomyocytes. *Am J Physiol Heart Circ Physiol* (2004) 287(3):H1149-59. doi: 10.1152/ajpheart.00060.2004. PubMed PMID: 15117716.

7. Tanaka H, Masumiya H, Sekine T, Kase J, Kawanishi T, Hayakawa T, et al. Involvement of Ca^{2+} waves in excitation-contraction coupling of rat atrial cardiomyocytes. *Life Sci* (2001) 70(6):715-26. PubMed PMID: 11833720.

8. Kirk MM, Izu LT, Chen-Izu Y, McCulle SL, Wier WG, Balke CW, et al. Role of the transverse-axial tubule system in generating calcium sparks and calcium transients in rat atrial myocytes. *J Physiol* (2003) 547(Pt 2):441-51. doi: 10.1113/jphysiol.2002.034355. PubMed PMID: 12562899.

9. Woo SH, Cleemann L, Morad M. Ca^{2+} current-gated focal and local Ca^{2+} release in rat atrial myocytes: evidence from rapid 2-D confocal imaging. *J Physiol* (2002) 543(Pt 2):439-53. PubMed PMID: 12205180.

10. Greiser M, Kerfant BG, Williams GS, Voigt N, Harks E, Dibb KM, et al. Tachycardia-induced silencing of subcellular Ca^{2+} signaling in atrial myocytes. *J Clin Invest* (2014) 124(11):4759-72. doi: 10.1172/JCI70102. PubMed PMID: 25329692.
